# Preclinical Testing of a New Dry Powder Aerosol Synthetic Lung Surfactant Formulation and Device Combination for the Treatment of Neonatal Respiratory Distress Syndrome

**DOI:** 10.1089/jamp.2025.0001

**Published:** 2025-08-06

**Authors:** Worth Longest, Michael Hindle, Dale Farkas, Mohammad A.M. Momin, Caleb Dalton, Felicia Hall, Ghali Aladwani, Hattie KenKnight, Robert M. DiBlasi

**Affiliations:** ^1^ Department of Mechanical and Nuclear Engineering, Virginia Commonwealth University, Richmond, Virginia, USA.; ^2^ Department of Pharmaceutics, Virginia Commonwealth University, Richmond, Virginia, USA.; ^3^ Center for Respiratory Biology and Therapeutics, Seattle Children’s Research Institute, Seattle, Washington, USA.

**Keywords:** aerosol surfactant therapy, infant aerosol therapy, neonatal RDS, synthetic lung surfactant, surfactant replacement therapy

## Abstract

**Background:**

This study advanced the preclinical development of a new dry powder aerosol synthetic lung surfactant (SLS) product for neonatal respiratory distress syndrome (RDS) by integrating a multiple-actuation device and scalable spray-dried formulation, evaluating physicochemical and *in vitro* aerosol performance, and then comparing biological efficacy with the current clinical standard of high-volume liquid bolus instillation.

**Methods:**

A new high-dose air-jet dry powder inhaler was developed that was characterized by a variable-volume aerosolization chamber (D3 device) with the goal of unifying aerosol quality and emitted dose (ED) over multiple actuations. The SLS excipient enhanced growth dry powder formulation was advanced through production on a scalable nozzle-based spray dryer system (Mini Spray Dryer; MSD2 formulation). Physicochemical characterization of the formulation was performed along with *in vitro* aerosol testing of the new D3-MSD2 device and formulation combination. The optimized D3-MSD2 aerosol therapy was then evaluated in a rabbit model of severe RDS.

**Results:**

The new D3-MSD2 combination produced a small-particle aerosol with high fine particle fraction (FPF_<5 µm_ = 87.9%; FPF_<2.5 µm_ = 61.6%) and percent ED (77.4% of loaded). Additional *in vitro* testing highlighted consistent particle size (D_v50_ = 1.6 µm) and ED across multiple actuations. In the animal model experiments, a total device-loaded formulation mass of 60 mg (delivered as 2x30 mg) produced a total phospholipid (PL) dose of 24 mg-PL/kg and a device ED of 18 mg-PL/kg compared with the 200 mg-PL/kg clinical dose of Curosurf liquid. *In vivo* response rate for the D3-MSD2 aerosol therapy was considerably more rapid with arterial oxygenation recovering 5–12 times faster than for liquid Curosurf. Biological response for the D3-MSD2 aerosol therapy was also superior with 2-fold improvement in final lung compliance compared with liquid Curosurf.

**Conclusions:**

The new D3-MSD2 aerosol therapy was found to be superior to clinical-practice liquid bolus instillation in the critical areas of required dose (order-of-magnitude reduction), delivery time, biological response rate, and efficacy.

## Introduction

Aerosol delivery of surfactant replacement therapy to treat neonatal respiratory distress syndrome (RDS) has been proposed as a relatively noninvasive technique that can avoid many of the challenges and risks associated with the current clinical practice of intubation beyond the vocal cords and intratracheal instillation of a large volume bolus of surfactant in carrier liquid.^1–4^ While surfactant aerosol delivery during noninvasive ventilation (NIV) is an ultimate goal,^1^ surfactant replacement therapy via aerosol to intubated subjects is also an important therapeutic target in neonatal RDS considering that (1) while NIV is recommended when possible, a significant fraction of the smallest and sickest preterm neonates will likely already be on invasive mechanical ventilation support^5,6^; (2) the process of liquid bolus instillation carries a number of peridosing risks and side effects including acute deterioration from liquid obstruction of the trachea and conducting airways, significant impairment in pulmonary gas exchange (frequently requiring resuscitation), bradycardia, pneumothorax, hemodynamic instability, and reduced cerebral blood flow^4,7–10^; and (3) multiple animal model studies indicate that liquid bolus delivery results in a nonuniform lung distribution, reduced compliance, heterogeneity in regional ventilation and perfusion, and a higher degree of alveolar structural damage and/or inflammation compared with aerosol surfactant therapy (AST).^11–16^ An additional finding from most previous animal studies that track oxygenation and lung biomechanics over time is that the response rate to liquid-instilled surfactant is often much faster than with aerosol delivery.^17–19^ The rapid PaO_2_ response may be clinically important considering ventilator-induced lung injury is continually occurring as a result of biomechanical forces, atelectasis, and inflammation that are all exacerbated in the absence of surfactant.^20^ As an intermediate step in the development of a truly noninvasive aerosol lung surfactant therapy, there may be substantial value in an aerosol therapy that can be easily delivered to intubated infants as needed, avoid the common side effects of administration by surfactant bolus liquid instillation, and prevent the associated alveolar damage that has been observed in preterm infants following concurrent liquid bolus instillation and mechanical ventilation.^21,22^ Through rapid aerosol delivery and targeting of aerosol deposition to the alveolar region, improved response to surfactant products can perhaps be achieved, including reduced required surfactant dose, significantly accelerated response time, and reduction in the duration of ventilation. Furthermore, product and dosage costs can be significantly reduced if an aerosol synthetic lung surfactant (SLS) product can be developed that works as well as or better than an animal-derived surfactant administered with liquid bolus instillation.^1,23,24^

In previous animal model and human subject studies that have directly compared AST with the clinical approach of liquid bolus instillation, disadvantages of aerosol products have often included:

1. higher administered dose for an adequate therapeutic response,
2. longer delivery times,
3. equivalent or slower response times, and
4. muted biological response at similar or increased aerosol doses, as reviewed below.

Considering the need for a higher required dose of the active phospholipid (PL) components when delivered as an aerosol, instilled liquid bolus products typically administer 100–200 mg-PL per infant kg of body weight (mg-PL/kg).^5,25^ In comparison, recent animal model and human subject studies with aerosol surfactants have administered 100–800 mg-PL/kg.^1,5,14,17,19,26–32^ Theoretically, ∼10 mg-PL/kg in the alveolar region is required for effective surfactant function in a preterm neonate,^33–36^ typically based on the formation of an approximate monolayer of PLs, which is consistent with surfactant levels recovered in lung lavage studies of healthy ∼1.5 kg animal models (typically 15–25 mg-PL/kg).^37^ Based on the required surfactant replacement therapy dose levels, for both instillation and aerosol administration, the percentage of PL that actually reaches the alveolar region appears to be very low, which is further supported by imaging studies.^38,39^

After intubation, surfactant delivery by liquid bolus instillation requires ∼10–15 minutes to complete.^40^ In comparison, liquid nebulized AST typically requires 30 minutes to 2.5 hours.^17,27^ The high-dose ARCUS® dry powder aerosol reduced the delivery time to 15 minutes in an animal model but required ∼400 device actuations timed with infant respiration and produced a muted biological response.^19^ In summary, AST typically requires a substantially longer administration time and higher dose compared with liquid bolus instillation.

Response rate to surfactant replacement therapy is often evaluated in animal models based on the recovery of oxygenation (or blood gases) and lung biomechanics metrics over time, either after surfactant administration or at the start of AST. Consistently, response time to AST has been either slower than or equivalent to liquid bolus instillation.^11,18,19^ Often, liquid bolus instillation provides a faster initial response in PaO_2_ (albeit at the cost of higher PaCO_2_ and ventilatory pressure requirements than aerosol^16^) and then blood gas levels and lung biomechanical metrics continue to recover over a period of 2–3.5 hours.^17,19,37,41^ The response rate is also significantly impacted by the level of lung damage in the RDS model and the use of positive-pressure lung inflations, typically associated with mechanical ventilation^37^ or positive-pressure resuscitation.

Considering biological response, several studies have reported similar or muted efficacy with AST compared with liquid bolus instillation. For example, in the recent nebulizer-based study of DiBlasi et al.^18^ with a rabbit surfactant washout model of RDS, identical aerosol and instilled doses of Alveofact produced similar biological improvements in most metrics considered. Moreover, final PaO_2_ on 50% oxygen did not exceed 175 mmHg, and lung compliance was reduced from pretreatment baseline at the time point of 180 minutes after AST. Considering dry powder aerosol delivery^19^ using ARCUS particle technology at a powder dose of 240 mg/kg and a similar rabbit surfactant washout model, blood oxygenation and airway compliance values only recovered to 63% (approximate final arterial oxygenation = 300 mmHg on 100% oxygen) and 54% (final compliance = 0.49 mL/cmH_2_O) of prewashout values, which were well below and slower than observed with liquid bolus instillation.

While efficient aerosol delivery to preterm infants is challenging,^42–47^ we propose that many of the limitations of current AST can be overcome, and high-efficacy performance versus liquid bolus instillation realized, if a dry powder surfactant aerosol with high activity can be rapidly and efficiently delivered to the deep lung regions, and preferably, all the way to the alveoli. To evaluate this theory, we are currently developing a new dry powder aerosol SLS product, which can accommodate aerosol administration through endotracheal tube (ETT), transnasal direct-to-infant, and NIV interfaces.^11,48,49^ As previously described,^48,49^ key components of this aerosol SLS product include the use of the small-particle aerosol excipient enhanced growth (EEG) delivery and targeting strategy,^50,51^ a dry powder micrometer-sized SLS-EEG formulation,^48^ and an infant dry powder air-jet aerosol delivery system (iDP-ADS).^52,53^ The lead SLS-EEG formulation currently contains pharmaceutical-grade PLs (dipalmitoyl phosphatidylcholine [DPPC] and 1-palmitoyl-2-oleoyl-sn-glycero-3-phospho-(1′-rac-glycerol) (sodium salt) [POPG]), a synthetic peptide mimic of surfactant protein B (B-YL),^24,54^ hygroscopic excipients (d-mannitol [MN] and sodium chloride [NaCl]), and a dispersion enhancer (l-leucine). A key component of the iDP-ADS is aerosol generation using an air-jet (AJ) aerosolization engine,^55,56^ and the previous study of Momin et al.^48^ employed a version with a fixed volume aerosolization chamber and overhanging outlet capillary (Design 2; D2) that performed well with up to 20 mg loaded powder doses. As reported by Momin et al.,^48^ combination of the D2 device and a specific SLS-EEG formulation (produced with the Buchi Nano Spray Dryer B-90 HP; resulting in the Nano Spray-Dried or NSD1 formulation) produced an emitted dose (ED) of >80% (loaded) and generated an aerosol with a mass median aerodynamic diameter (MMAD) <2 µm. Using the *in vitro* characterization data, including delivery to a realistic *in vitro* rabbit airway model, a dose delivery protocol or profile (DDP) was designed in which a 60 mg total device-loaded formulation mass (containing 24 mg-PL/kg) would be administered and deliver an approximate lung dose of 15–18 mg-PL/kg with a total aerosol delivery period <5 minutes.

The companion study of DiBlasi et al.^11^ evaluated the performance of the D2-NSD1 device and formulation combination and DDP designed by Momin et al.^48^ in an intubated rabbit model of severe RDS induced by surfactant washout and compared response with liquid bolus instillation of Curosurf (200 mg-PL/kg). The total loaded dose of the dry powder SLS-EEG NSD1 formulation (60 mg containing 24 mg-PL/kg) was administered over multiple dose delivery cycles due to the 20 mg maximum limit of the D2 device (for optimal performance). Despite the order of magnitude lower PL dose of the aerosol product compared with Curosurf, the relatively high lung transmission of the aerosol beyond the upper tracheobronchial (TB) airways,^48^ and possibly deeper within the lungs, resulted in near-complete recoveries of arterial oxygenation (PaO_2_; 96%–100% recovery) and oxygenation index (OI) over the 3.5-hour monitoring period, with a recovery rate that was very similar to Curosurf administered with liquid bolus instillation. Importantly, the aerosol group had superior recovery of lung compliance compared with liquid-instilled Curosurf, as well as less histological evidence of lung tissue injury, greater uniformity in lung aeration, and more uniform distribution of surfactant in the lung parenchyma. Total delivery time of the aerosol was ∼5 minutes; however, total treatment time including device reloading and animal stabilization on the ventilator was ∼20 minutes.

While the *in vivo* response to the previous D2-NSD1 combination was excellent at an order of magnitude less PL dose compared with current clinical practice,^11,48^ some limitations related to the device and SLS-EEG formulation were identified. Considering the performance of the device, acceptable aerosolization limited the powder mass loading to 20 mg per dose cycle, which extended the total treatment time and required several otherwise unnecessary cycles of disconnecting and reconnecting the mechanical ventilator. With each 20 mg loaded dose, the D2 device was only actuated ∼4 times; however, inconsistent emptying of the device was observed across these four actuations, with a majority of the powder mass emptying on the first actuation.^57^ Overly rapid emptying of the powder resulted in an excessively dense aerosol cloud, potentially leading to an aerosol fine particle fraction (FPF) <5 µm that was relatively low at ∼30% of the ETT ED. Finally, the response rate to the administered aerosol appeared more consistent with mid-level TB deposition of the aerosol followed by subsequent slow spreading of the surfactant to the alveoli, whereas true alveolar deposition of a majority of the dose may theoretically provide a very rapid recovery of lung function.

Considering the formulation, the NSD1 dry powder was spray-dried using the Buchi Nano system, which employs a unique mesh nebulizer design and electrostatic precipitator to collect the powder.^58^ These unique elements of the Buchi Nano system are advantageous for forming very small primary particles; however, they represent a laboratory scale approach, which is inconsistent with the two-fluid nozzle system used in most industrial scale spray dryers. Furthermore, spray drying with the Buchi Nano was found to degrade ∼50% of the B-YL peptide, possibly in the electrostatic precipitator, which is currently the most expensive component of the formulation despite its low concentration.

This study builds on our previous work^11,12,48,49,52,59–63^ toward the development of a dry powder AST with the inclusion of a new next-generation air-jet dry powder inhaler (DPI) device and formulation combination for administration to preterm infants via an ETT interface. Specifically, the objective of this study was to integrate the new variable-volume chamber AJ DPI and scalable spray-dried formulation combination into the iDP-ADS, perform physicochemical characterization of the formulation together with aerosol formation and *in vitro* testing, and then evaluate biological efficacy of the resulting AST in a preterm infant-size animal model of RDS. The new device, termed the D3 AJ DPI, employed a variable-volume aerosolization chamber design, which was intended to enable higher drug mass loadings and better unify dose emission per actuation, potentially improving aerosol quality at high powder mass loadings. The new SLS-EEG formulation was produced on a scalable nozzle-based spray drying system (Buchi S-300 Mini Spray Dryer) and termed the SLS-EEG MSD2 formulation. Quality of the formulation was first evaluated based on imaging and physicochemical analysis. Aerosolization performance of the new device and formulation combination was then determined using *in vitro* assessment, including cascade impaction, laser diffraction sizing, and testing in a realistic airway model. Initial goals for aerosol quality included maximizing FPFs with cutoffs <5, <2.5, and <1 µm to the extent possible compared with the previous D2-NSD1 combination (with FPF_<5 µm_ ∼30% of ED) and achieving MMAD and median volumetric diameter (D_v50_) values in the range of ∼1.5–2.0 µm. Once aerosol quality and ED metrics were achieved, *in vivo* performance of the new D3-MSD2 aerosol therapy was established in an intubated rabbit model of severe RDS with a body weight consistent with preterm infants at a gestational age (GA) of 31 weeks (∼1.5 kg). Finally, *in vitro* data and *in vivo* responses were compared to determine DDP metrics leading to a high-efficacy response, in terms of both required active (PL) dose and recovery response rate.

## Materials and Methods

### Formulation materials

PLs (DPPC and POPG) and surfactant protein mimic peptide (B-YL) were purchased from Avanti Polar Lipids, Inc. (Alabaster, AL) and CSBio (Menlo Park, CA), respectively. l-leucine was purchased from Sigma-Aldrich Chemical Co. (St Louis, MO). Growth excipients NaCl and MN together with other chemicals were purchased from Fisher Scientific Co. (Hanover Park, IL). Throughout the study, freshly collected deionized water was used.

### Formulation production and analytical methods

Multiple batches of the micrometer-sized SLS-EEG MSD2 formulation were produced using the two-fluid spray nozzle-based Buchi S-300 Mini Spray Dryer system (Büchi Labortechnik AG, Flawil, Switzerland). Briefly, the feed dispersions were prepared with a solids composition of DPPC 55%, POPG 7.5%, surfactant protein B peptide mimic B-YL 2.5%, mannitol 9%, sodium chloride 6%, and l-leucine 20% w/w in an ethanol-water (5:95% v/v) cosolvent system, which was sonicated for 40 minutes in a heated water bath (45°C–55°C) with intermittent manual shaking every 10 minutes.^60,64^ The resulting PL (DPPC and POPG) and B-YL nominal content of the MSD2 formulation were 62.5% and 2.5%, respectively, compared with the previous NSD1 formulation with corresponding theoretical values of 65% and 5%, respectively. The total solids content of the MSD2 feed dispersion was 0.125% w/v. During spray drying, the feed dispersion was continuously stirred using a magnetic stirrer. Spray drying conditions included the use of a 0.5 mm spray nozzle, drying gas and spray gas flow rates of 30 m^3^/hr and 1200 L/hr, respectively, drying temperature of ∼80°C, and an outlet temperature of ∼45°C–46°C. Dried powder formulations were collected in a high-efficiency cyclone with a backup bag filter. Immediately postcollection, powder formulations were equilibrated in a glass vial (∼17% relative humidity [RH] at ∼24°C for 30 minutes) and then stored in a sealed desiccator at 0% RH/−20°C until needed for use.^64^

Validated high-performance liquid chromatography (HPLC) methods were used to quantify DPPC mass in determining aerosol size distribution from cascade impaction and lung delivery efficiency from the *in vitro* lung chamber (LC) model. The HPLC system consisted of an Alliance 2695 Separation Module with a photodiode array ultraviolent (UV) detector and Empower 2 data acquisition software (Waters Corporation, Milford, MA). The sample injection volume was 100 µL and column temperature was 45°C. The chromatographic separation was achieved using the Waters XSelect CSH C18 XP (2.5 µm, 4.6 × 100 mm; Waters Corporation, Milford, MA). Further details including the descriptions of the gradient elution system and UV absorbance, as well as a separate method using a similar approach for the quantification of B-YL used in content uniformity testing, were previously reported in Momin et al.^48^

### Laser diffraction particle sizing

Powder dispersibility and primary particle size of the SLS-EEG MSD2 formulation were determined using laser diffraction at multiple dispersion pressures. Specifically, components employed included the Sympatec HELOS (Sympatec GmbH, Germany) equipped with a RODOS/M disperser and an ASPIROS sample feeder. A small glass vial was filled with ∼3 mg of spray-dried powder, placed in the sample feeder, and dispersed in the laser beam using the RODOS/M disperser with an R1 lens at dispersion pressures of 0.5 and 4.0 bar. The particle size distribution results (Dv_10_, Dv_50,_ and Dv_90_) were obtained using Sympatec WINDOX 5.10 software (Sympatec GmbH, Germany).

### Powder imaging

The particle surface morphology of the SLS-EEG MSD2 formulation was evaluated using a Hitachi SU-70 scanning electron microscope (SEM; Hitachi High-Tech, Tokyo, Japan) at an accelerating voltage of 5.0 kV. A small amount of powder sample was mounted on a metal SEM stub using double-sided carbon tape, and loose particles were removed using compressed air. The samples were sputter coated with platinum grain using a Denton Desk V sputter coater (Denton Vacuum, Moorestown, NJ) before imaging. Aperture, astigmatism (X&Y), and beam alignment were adjusted for optimal imaging, and then the image was captured at 5 kV with a 5 mm working distance.

To capture internal particle structure, a combination of focused ion beam (FIB) cutting and SEM imaging approach was employed through the use of a Scios 2 DualBeam system (ThermoFisher Scientific, Waltham, MA). Once the sample preparation was completed as described above, the specimen was fixed in the sample holder and then inserted into the FIB chamber. Contrast and brightness, astigmatism (X&Y), and focus were adjusted to identify an area of interest using the SEM mode. Then, in FIB mode, the sample was positioned at a 7 mm working distance from the electron beam and tilted at 52° with horizontal. The FIB column was set to 30 kV with ion beam current of 10 pA to mill the particles while minimizing damage to the internal structure, and then the SEM mode was activated to capture the image at 5 kV with a T2 detector setting.

### X-ray powder diffraction and moisture sorption testing

The Rigaku Miniflex 6G (Rigaku, Japan) X-ray powder diffraction (XRPD) technique was used to characterize the SLS-EEG MSD2 formulation. Briefly, a small amount of powder was placed on the center of the XRPD sample holder and then the powder surface was gently smoothed using a glass slide. The samples were scanned using a continuous mode in the range of 3°−50° 2θ at a scan speed of 1.5°/min.

To understand the water uptake of the SLS-EEG MSD2 formulation across a wide range of RH values, samples were exposed using the dynamic vapor sorption system (DVS Adventure, Surface Measurement Systems Ltd., UK). Briefly, ∼5mg of powder sample was equilibrated at 0% RH and then exposed to increasing RH from 0% to 95% (sorption cycle) and decreasing RH from 95% to 0% (desorption cycle). At each RH, once the defined equilibrium condition (the rate of mass change [dm/dt] <0.002%) was achieved, the RH was changed to the next level.

### Aerosol delivery system and D3 AJ DPI

**FIG. 1.**
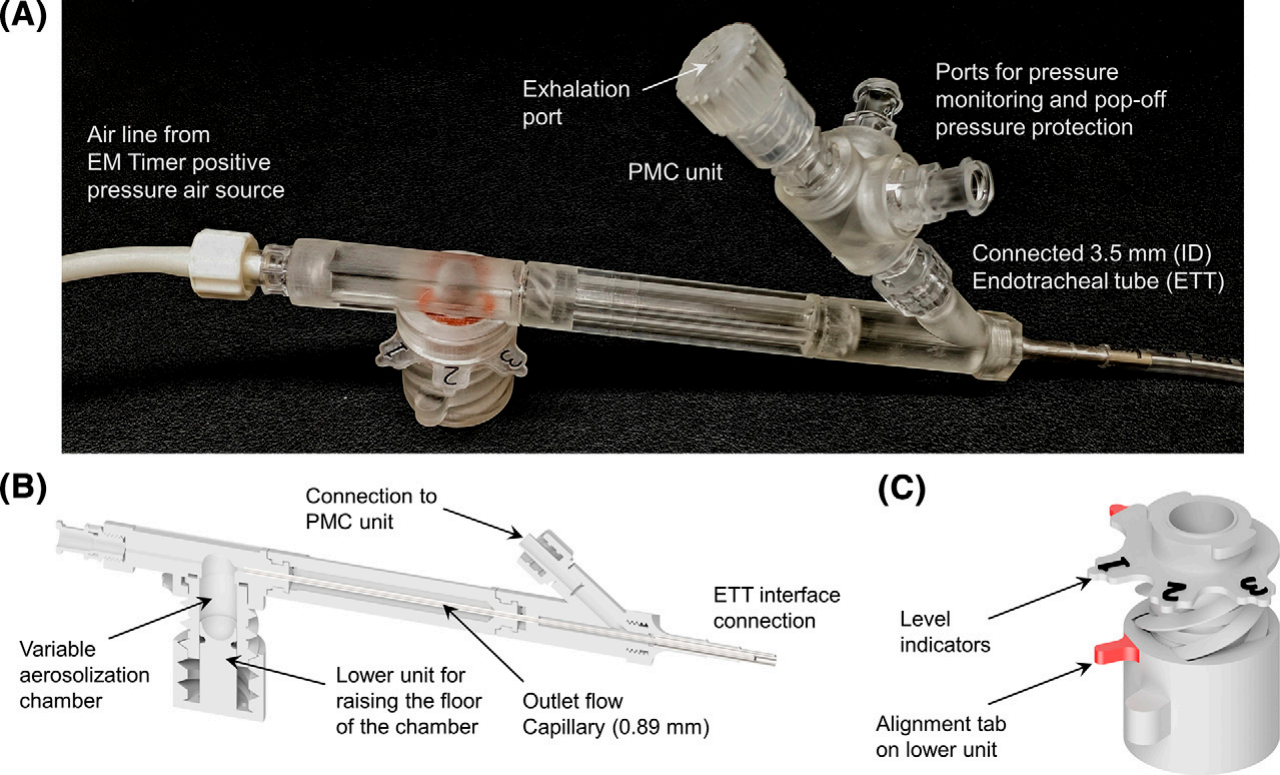
Components of the infant dry powder air-jet aerosol delivery system (iDP-ADS) including **(A)** setup of the D3 air-jet DPI with connection to the electromechanical (EM) Timer air source, pressure monitoring and control (PMC) unit, and endotracheal tube (ETT) interface connector, **(B)** inner flow pathway for the D3 air-jet DPI with variable aerosolization chamber, and **(C)** model of the variable aerosolization chamber components including lower unit level indicators. ID, internal diameter.

The iDP-ADS used in this study consisted of an electromechanically controlled timer air source (EM Timer), aerosolization engine, pressure monitoring and control (PMC) unit, and ETT interface connector (Fig. 1). Considering the EM Timer, the inlet pressure regulator was set to provide a 3 L/min flow rate to the device of compressed air (*in vitro* testing) or pure oxygen (*in vivo* testing). The timer setting was adjusted to deliver a gas actuation volume (GAV) of 10 mL during *in vitro* testing and ∼7 mL/kg for the *in vivo* experiments. In the animal model experiments, a table was used to ensure that for the measured lung compliance of individual animals, the delivery of 7 mL/kg would result in an elastic lung pressure rise (ΔP_E_ = 1/C*GAV, where C is the lung compliance in milliliters per centimeters of water) ≤ 20 cmH_2_O.^48^ If the ΔP_E_ was greater than the 20 cmH_2_O limit, then the GAV was reduced to meet the pressure limit, at the discretion of the administering clinician. Further details of this procedure, and the corresponding lookup table, were provided in the previous study by Momin et al.^48^

The PMC unit enabled the control of respiration, pressure monitoring, and pressure protection during the *in vivo* experiments and *in vitro* LC testing. A flow control orifice (0.5 mm diameter) was used in the exhalation port to slow exhalation and better enable the administrator to maintain a positive end expiratory pressure (PEEP) of ∼5 cmH_2_O (Fig. 1A). Additional ports on the PMC unit led to an analog pressure gauge (AG Industries, St. Paul, MO) and pressure relief valve, adjusted to ∼25 cmH_2_O. For device actuation, at a lung pressure of ∼5 cmH_2_O, the administering clinician covered the exhalation port, creating an airtight seal with the lungs, and actuated the device. The exhalation port remained covered for ∼1 second. The administering clinician then uncovered the exhalation port and monitored the lung pressure during exhalation until ∼5 cmH_2_O was achieved, at which point the process was repeated. Approximately four device actuations were used, accompanied by a defined rotation of the variable-volume chamber lower unit after each actuation, for each 30 mg device-loaded dose.

A primary component of the iDP-ADS was the infant AJ DPI (Fig. 1B). As previously described, AJ DPIs are designed with geometrically simple elements (small diameter flow passages and smoothly curving aerosolization chamber) and engineered to enable the efficient generation of small-particle aerosols with limited volumes of air.^55,56,65–67^ In previous studies, the aerosolization chamber has been of fixed dimensions and volume, which for the SLS-EEG and other surfactant formulations limited device loading to ≤20 mg to maintain high quality aerosolization,^48,59^ in the absence of a cyclic loading mechanism.^52,68^ Higher mass loadings either emitted the aerosol too quickly, degrading aerosol quality, or had reduced ED. In this study, we introduce a new variable-volume aerosolization chamber with a movable lower unit, which controls the floor height of the chamber (Fig. 1B, C). For loading of high powder masses, the floor of the aerosolization chamber is positioned at the lowest level, maximizing the available chamber volume. With a first actuation, the geometry is designed such that secondary air currents aerosolize a defined mass of powder on the top of the powder bed while leaving the remaining powder relatively undisturbed. The floor of the variable-volume chamber is then raised by a defined distance using a small turn of the lower unit (Fig. 1C), exposing the next dose of powder for aerosolization with the next actuation. By following an actuate-and-turn sequence, the dose per actuation can potentially be unified while enabling high powder mass loading and maintaining high aerosol quality. As illustrated in Figure 1B, alignment of the positioning tab on the lower unit with the numbers indicating levels of the aerosolization chamber floor was used to guide rotation of the lower unit through four defined levels. During *in vitro* testing, one actuation per level was employed. During *in vivo* delivery, one actuation per level was also used; however, the operator could employ additional actuations at each level with minimal powder emissions if needed to maintain animal ventilation.

### Aerosol size characterization using cascade impaction

The aerosol emitted from the D3 AJ DPI with the ETT adaptor attached was characterized using cascade impaction with a next-generation impactor (NGI; MSP, TSI Incorporated, Shoreview, MN). The D3 device was connected to the preseparator inlet of the NGI, as previously illustrated by Howe et al.,^52^ using a custom adapter. The NGI was oriented at 90° to horizontal to enable the device to remain in the intended level orientation for use, and the custom adaptor positioned the ETT adapter of the D3 device in the center of and ∼1 cm away from the preseparator inlet. Side openings in the custom adapter enabled the entrainment of room air around the aerosol plume, allowing the NGI to be operated at a constant flow rate of 45 L/min, generated from a downstream vacuum source. The NGI flow rate of 45 L/min was chosen to ensure collection of the aerosol, minimize any effects of settling, and provide appropriate stage cutoff diameters for evaluating small aerosol sizes. Each stage of the NGI was coated with MOLYKOTE® 316 silicone spray (Dow Corning, Midland, MI) to minimize particle bounce and re-entrainment. Before each set of experimental runs, the flow rate was confirmed using a flow sensor (Sensirion SFM3000, Sensirion AG, Stafa, Switzerland) connected to the NGI inlet.

For aerosol size determination, the D3 device was loaded with 30 mg of the MSD2 formulation. Consistent with aerosol delivery to a 1.5 kg infant, 10 mL GAVs were employed at 3L/min, which required an actuation time of ∼0.21 seconds. One actuation was used for each height of the variable-volume chamber device across four levels (three marked as shown in Fig. 1C with a final top level at the maximum floor height). Preliminary development experiments were conducted to determine the correct chamber volume and rate of floor rise to efficiently deliver a high-quality aerosol and maintain a high ED for a 30 mg device-loaded formulation mass and four device actuations, resulting in a target powder mass per actuation of ∼5–10 mg. Three replicate runs for each experiment were performed, and quantitative analysis of DPPC deposition using the HPLC-UV assay was used to assess the aerosol performance characteristics of the SLS-EEG powder formulation. Analysis metrics included ED, MMAD, FPF, and geometric standard deviation. Percent ED (%ED) was calculated as the mass of DPPC in the loaded dose minus the mass of DPPC remaining in the device divided by the initial loaded mass of DPPC (×100 for %ED). Aerosol size calculations were based on the mass of DPPC recovered in the NGI (including preseparator). Based on an airflow rate of 45 L/min, the NGI stage cutoff diameters were determined using the formula specified in USP 35 (Chapter 601, Apparatus 5). The MMAD and FPFs (<5 µm, <2.5 µm and <1 µm) were calculated using Copley Inhaler Data Analysis Software (CITDAS).

### Characterization by actuation using SprayVIEW, Spraytec, and emitted mass

A combination of light sheet microscopy (Proveris Scientific SprayVIEW®), laser diffraction particle sizing (Malvern Spraytec®), and gravimetric analysis was used to characterize aerosolization performance on the basis of individual actuations. For each experiment, 30 mg of the MSD2 formulation was loaded into the D3 device, and the device was actuated one time at each level of the variable-volume chamber, denoted as actuations A1–A4, using 10 mL GAVs delivered at 3 L/min from the EM Timer. All experiments were completed in triplicate, denoted as runs R1-R3.

With the SprayVIEW system, the device outlet capillary was placed at the furthest position within the camera field of view, and the laser housing was adjusted to the center of the orifice in the vertical and horizontal directions. To simultaneously record gas flow rate, an inline flow meter (Sensirion SFM3400-AW and RS485-USB/2028) was connected between the EM Timer outlet and D3 device inlet. Image acquisition was set at a frame rate of 500 Hz over a 1-second time acquisition window. As with all *in vitro* cases, the EM Timer was actuated for 0.21 seconds, which generated the 10 mL GAV at a flow rate of 3 L/min. Analysis of the SprayVIEW data was used to report plume geometry, velocity, and intensity. While a quantitative correlation is not available between SprayVIEW-reported plume intensity and aerosol mass emission, intensity over actuation time was used as a qualitative metric of aerosol emission relative to device actuation flow.

To determine aerosol size distribution for each of the four actuations, the Malvern Spraytec laser diffraction system was employed in the default (open bench) configuration with 1 kHz data acquisition rate and employed a 300 mm lens (0.1–900.0 µm nominal size range). Refractive indices were selected as standard opaque particles (1.50 + 0.50i) sprayed into air (1.00 + 0.00i). Considering the density of the aerosol cloud, necessary system modifications included only considering detector ranges from 16 to “Last” and removal of plastic flanges to prevent cloud recirculation into dead zones. All other parameters remained under manufacturer default values. Concentrated-weighted, time-averaged aerosol polydisperse size distributions (PSDs) and median volumetric diameters (D_v50_) values were determined using the Malvern Spraytec 4.00 software for each of the four device actuations (A1–A4).

The emitted formulation mass for each actuation was determined in separate experiments in which the unloaded and loaded D3 device was weighed, using an Ohaus Analytical Plus (Model: AP250D) microbalance (Ohaus Corporation, NJ, USA), and then the loaded device was reweighed after each actuation. Following each actuation, the emitted mass for that actuation was determined as the difference between the previous loaded device mass and current device mass (in mg). %ED per actuation was calculated as the percentage of the emitted mass relative to the original loaded mass (loaded device mass − unloaded device mass). Total emitted mass and %ED were determined as the sum of the emitted masses over the four actuations and the percentage of this sum relative to the original loaded mass, respectively. In method validation experiments, gravimetric analyses of percent emptying matched HPLC quantification (to within 10% relative difference), further validating gravimetrically recorded mass losses for calculating %ED and emitted mass.

### In vitro testing of lung delivery efficiency with the LC model

**FIG. 2.**
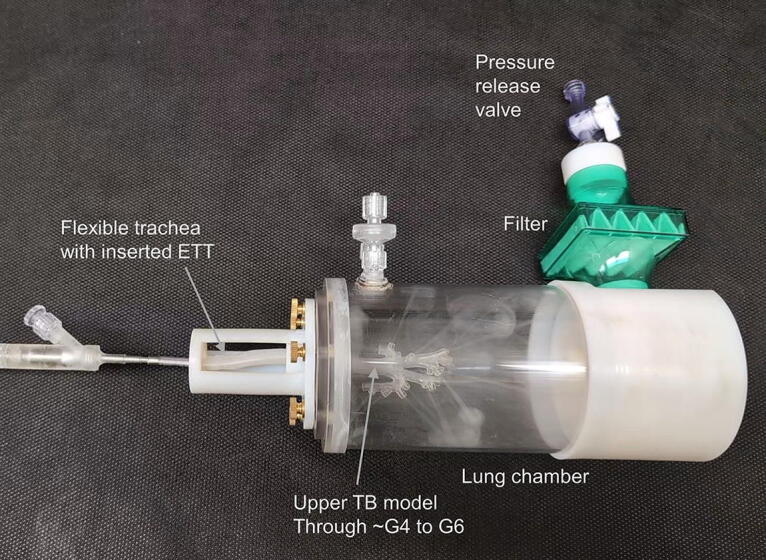
Actuation of the D3-MSD2 device and formulation combination into the *in vitro* lung chamber (LC) model including an upper tracheobronchial (TB) region consistent with a ∼1.5 kg rabbit. The time point shown is just after the second actuation visualizing a standing cloud of aerosol in the chamber (from actuation A1) with the addition of incoming jets of aerosol flow from the upper TB model outlets associated with A2. G#, airway generation number.

To approximate the lung delivery efficiency achieved in the *in vivo* animal model experiments, a realistic rabbit LC model was employed, as previously described by Momin et al.^48^ (Fig. 2). Briefly, upper airway aerosol transport and deposition was captured using a 3D-printed model of the upper TB airways extending from the trachea to outlet generations from approximately G4 to G6, which was extracted from a computed tomography scan of a small mammal with body mass, airway structure, and dimensions very similar to young mature New Zealand White Rabbits.^48^ Outlet diameters of the upper TB region ranged from 0.8 to 2.3 mm. A majority of the trachea was constructed in flexible silicone material to enable realistic insertion of the ETT with airway sealing. The upper TB model was surrounded by a cylindrical LC, with a total sealed system volume of ∼500 mL, resulting in a realistic measured compliance of 0.45 mL/cmH_2_O, consistent with preterm infants experiencing RDS. A filter (Pulmoguard II™, Queset Medical, North Easton, MA) and pressure release valve were used to capture aerosol that did not deposit in the upstream components. To evaluate regional aerosol deposition in the LC model, the inner walls of the airway model were coated with MOLYKOTE 316 silicone spray (Dow Corning, Midland, MI) to minimize particle bounce and simulate an airway fluid coating. The D3 device was loaded with 30 mg of the SLS-EEG formulation and connected to the ETT, which was inserted into the flexible silicone trachea. The LC model was positioned horizontally on a flat surface, similar to the orientation of the rabbits in the *in vivo* experiments. The AJ DPI was actuated at 3 L/min with 10 mL GAVs one time at each of the four variable-volume chamber levels (Fig. 2), with the LC pressure monitored and released after each actuation. After removing the AJ DPI ETT connection, a final pressure chamber evacuation was performed to capture any remaining suspended aerosol on the filter using a downstream vacuum pump. Deposited powder formulation was recovered from the device, ETT, upper TB model, chamber, and filter using appropriate wash volumes of methanol, and those solutions were analyzed by HPLC-UV to determine the DPPC content. The chamber and filter deposition fractions were grouped together as a lower lung deposition fraction. The upper TB, chamber, and filter deposition fractions were grouped together as a total lung deposition fraction. All deposition fractions were expressed as a percentage of device nominal loaded dose based on the HPLC quantification of DPPC and were calculated from loaded powder mass and previously determined DPPC content.

### In vivo surgical preparation and instrumentation

All experimental animal procedures were conducted according to the National Institute of Health’s Guide for the Care and Use of Laboratory Animals and approved by the Seattle Children’s Institutional Animal Care and Use Committee. Juvenile female New Zealand White Rabbits (Western Oregon Rabbit Company, Philomath, OR) were tranquilized, sedated, instrumented, monitored, and ventilated according to identical methods established in previously published animal studies.^11,18,69^ At the time of *in vivo* testing, body weight of the D3-MSD2 aerosol group was 1.59 ± 0.23 kg, and body weight of the entire cohort including sham and Curosurf liquid historical controls was 1.56 ± 0.07 kg.

**FIG. 3.**
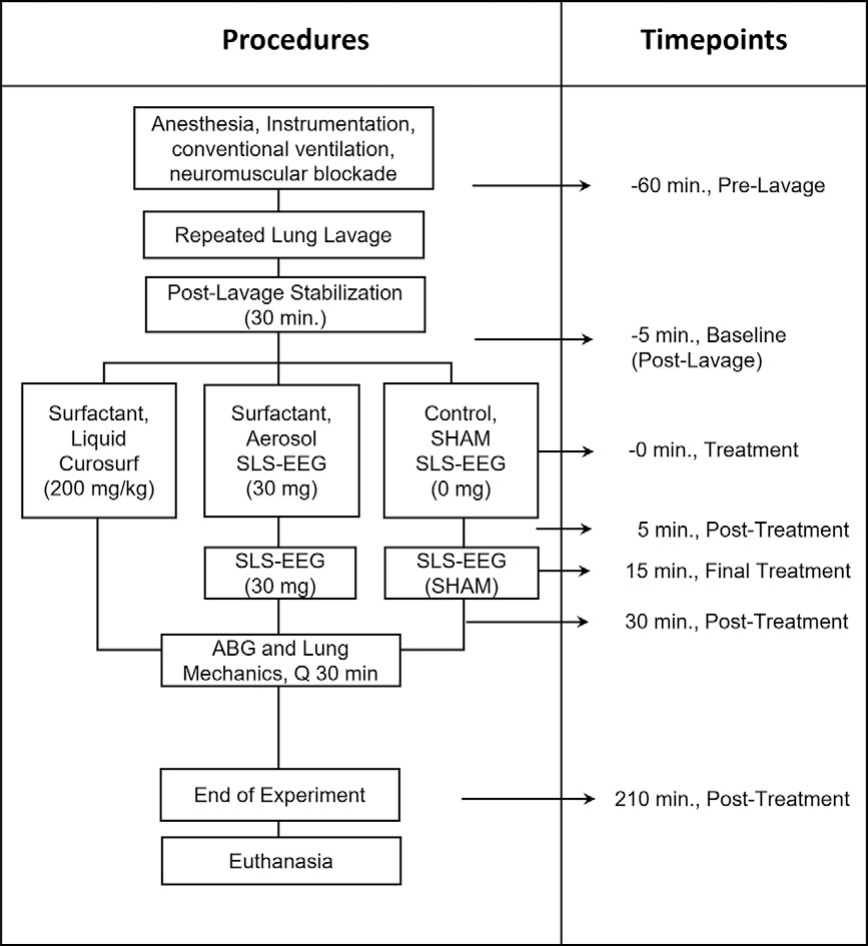
Time schedule for animal experimental procedures. The sequence of procedures for lung lavage to establish surfactant deficiency and lung injury, treatment groups, time schedule for procedures, surfactant dosing, and physiological measurements is shown. ABG, arterial blood gas; SLS-EEG, synthetic lung surfactant–excipient enhanced growth formulation.

Animals were administered rocuronium bromide to induce neuromuscular blockade during the lung lavage and surfactant administration. Subsequent rocuronium doses were withheld to promote spontaneous inspiratory efforts to assist mandatory ventilator breaths, consistent with the clinical management of preterm infants. Before lung lavage for baseline RDS condition, arterial blood gases (ABGs), lung mechanics, ventilation parameters, and vital signs (i.e., heart rate, blood pressure, and temperature) were acquired at a time point of −60 minutes, as summarized in Figure 3.

### In vivo induction of surfactant deficiency and lung injury

Severe lung injury was induced by a multiple lung insult (hit) model that included hyperoxia, atelectotrauma, extensive surfactant washout (typically 6–10 cycles), and mechanical ventilation.^11,19^ For injury induction, the FiO_2_ was increased to 1.0, and PEEP was reduced to 0 cmH_2_O. The lungs were depleted of surfactant and injured by cyclic alveolar collapse (PEEP of 0 cmH_2_O) with repeated lavage using 25 mL/kg of warmed (39°C) normal saline (0.9%), with 5-minute recoveries between lavages until the static lung compliance was 50% of the prelavaged value, SpO_2_ of 90%–92% on 1.0 FiO_2_ and a PEEP of 5 cmH_2_O.^69^ The animals were deemed surfactant deficient when PaO_2_ <100 mmHg was confirmed by two consecutive ABGs 30 minutes apart following lavage. Additional lavages were administered as needed to establish these surfactant depletion and lung injury goals. Following the establishment of surfactant deficiency and severe lung injury, the ventilator rate was initially titrated based on PaCO_2_ values of 55–65 mmHg and pH 7.25–7.35, and animals remained on these settings throughout the rest of the experiment. If pH <7.25 at any point, the respiratory rate was increased as needed. Animals with PaCO_2_ <40, pH >7.40, and evidence of alveolar overdistension (reduced compliance for the terminal 20% of inspiration, C_20_/C ratios <0.80)^70^ had the respiratory rate reduced to prevent pneumothorax.

### In vivo experimental protocol

The sample size for the experimental animal group (SLS-EEG) was calculated with a 95% confidence interval, a power of 90%, and a standard deviation (SD) of 0.20; the total recommended sample size was seven (*n* = 7) for the experimental group based on the mean and SD for PaO_2_ from a previous animal study that compared between two different surfactant aerosol delivery systems.^71^ The negative control (sham) and positive control (liquid Curosurf) groups included four and five animals in each group, respectively.

Figure 3 shows a diagram of the animal procedures, study time points during the experiments, and the DDP. Following surfactant washout, animals in the aerosol treatment group received MSD2 aerosol formulation from the iDP-ADS using the D3 device (*n* = 7). Animals receiving the D3-MSD2 combination aerosol therapy were compared with historical controls previously reported in DiBlasi et al.^11^ that received either a clinical dose of intratracheally instilled liquid surfactant (Curosurf at 200 mg/kg; *n* = 5) or the aerosol delivery process without powder (*n* = 4) as a (negative sham) control at time point 0. All subjects were supported for 3.5 hours following the initial intervention.

### Intratracheal liquid surfactant administration (Curosurf)

As reported in DiBlasi et al.,^11^ the animals in the liquid Curosurf control group received standard intratracheal administration of porcine-derived Curosurf surfactant (poractant alfa, Chiesi, Parma, Italy). Curosurf comprises ∼96% PLs (80 mg/mL) and 1% surfactant proteins B and C and is only approved for administration by tracheal liquid instillation. The vials of liquid Curosurf (200 mg/kg; 2.5 mL/kg) were drawn up into a syringe and administered based on a standard protocol, described in greater detail elsewhere.^9^

### Dry powder surfactant aerosol delivery

Animals in the D3-MSD2 aerosol group received 60 mg (device-loaded dose) dry powder formulation delivered directly to the ETT. The 60 mg total device-loaded formulation mass was divided into two dose cycles, each delivering 30 mg device-loaded formulation masses and administered 15 minutes apart (t = 0 and 15 minutes). This dose magnitude and delivery timing was selected for consistency with the previous study of DiBlasi et al.^11^ where we used the D2-NSD1 combination, in order to enable direct comparison of the *in vivo* responses and best isolate the *in vivo* impact of D3-MSD2 improvements in aerosol quality. With the updated MSD2 formulation, containing less B-YL, and a 1.59 kg average rabbit weight for the aerosol group, the initial total device-loaded PL dose was 24 mg-PL/kg, which is consistent with the 24 mg-PL/kg from the previous NSD1 formulation.^11,48^ It is noted that each 30 mg device-loaded formulation mass of MSD2 contains only 12 mg-PL/kg. Furthermore, the experimentally determined device %ED of ∼77.4% (NGI) from the ETT adaptor results in a device total emitted mass of 18 mg-PL/kg entering the ETT. Considering the Curosurf dose of 200 mg-PL/kg is also delivered directly to the ETT, we view the AST as delivering approximately one order of magnitude less active therapeutic (PL dose) to the animal.

**FIG. 4.**
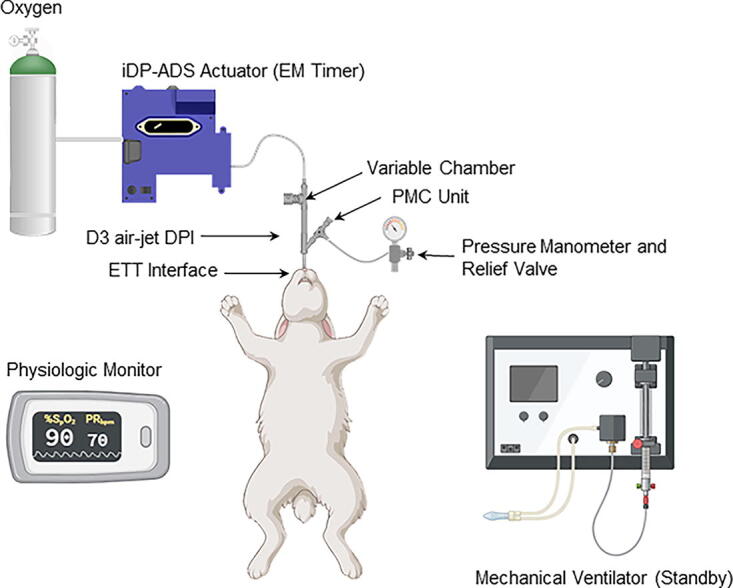
*In vivo* testing setup illustrating aerosol delivery from the iDP-ADS using the D3 air-jet DPI with the ETT interface. iDP-ADS, infant air-jet dry powder-aerosol delivery system; PEEP, positive end expiratory pressure; PIP, peak inspiratory pressure.

During aerosol delivery, the D3 AJ DPI was connected directly to the rabbit ETT, enabling precise control over the peak inspiratory pressure (PIP) of 25 ± 2 cmH_2_O, PEEP of 5 cmH_2_O, V_T_ of 7 mL/kg, flow of 3 L/min, and actuation time of ∼0.20–0.22 seconds with the iDP-ADS (see Fig. 4). The EM Timer settings for the DPI were determined based on the postlavage compliance values and weight (kg) using a reference calculation chart, which is described in greater detail in the study of Momin et al.^48^ A total of ∼4 actuations were employed to deliver each 30 mg device-loaded formulation mass. The iDP-ADS employed an adjustable pressure relief valve (∼25 cmH_2_O) and an analog pressure manometer, akin to a manual resuscitator, to measure pressure delivery and prevent barotrauma. The powder chamber was manually filled with formulation from previously packaged capsules (Qualicaps Inc, Whitsett, NC) that were stored in Al-Al blister packs, and the DPI was actuated to deliver the aerosol and inflate the lungs, followed by an ∼1-second breath hold, after which exhalation was allowed through the exhalation port. The animal was promptly returned to the ventilator following each of the two dosing cycles (with 30 mg device-loaded formulation mass each) conducted at *t* = 0 and 15 minutes.

### Physiological measurements

Figure 3 shows a diagram of the animal procedures, study time points during the experiments, and the DDP. Following lung lavage, gas exchange (ABGs), ventilation parameters, and hemodynamic measurements were obtained at the pretreatment (RDS baseline) condition at −5 minutes. Between −5 and 0 minutes, the initial D3-MSD2 aerosol (30 mg) dose or sham was delivered, or liquid instillation of the total dose of Curosurf was completed. Five-minute time point measurements were then taken to assess the short-term gas exchange effects immediately following therapy due to lung volume derecruitment from the initial ventilator disconnection with the D3-MSD2 aerosol therapy or sham delivery, potential airway obstruction from liquid Curosurf or powder agglomeration, as well as the potential for very rapid response to the AST. The D3-MSD2 aerosol group had two additional measurements at 10 and 15 minutes, the latter of which occurred just before the second 30 mg device-loaded dose. Standard posttreatment measurements were obtained at 30-minute intervals thereafter for 3.5 hours (see Fig. 3).

Respiratory rate, PIP, PEEP, and mean airway pressure were acquired from the ventilator. The pressure–volume relationship was evaluated by performing a brief inspiratory hold and recording the static compliance. The compliance was normalized based on the individual rabbit weights (milliliter per centimeter of water per kilogram). The OI and ventilation efficiency index (VEI) were calculated using ventilator parameters and ABG values to assess disease severity at each time point.^72–74^ Animals were euthanized with 100 mg/kg Euthasol following the 3.5-hour postsurfactant measurements and remained ventilated for necropsy.

### Statistical analysis

Statistical analysis of the *in vitro* data for comparing aerosol delivery performance and simulated lung delivery efficiencies was performed using JMP Pro 17 (SAS Institute Inc., Cary, NC). For *in vivo* data, results were calculated as mean ± standard error of the mean for all continuous physiological variables at each time point for surfactant-treated and control groups. Two-way analysis of variance was used to assess the treatment effects of each group and time point, as well as their interactions. Tukey’s multiple comparisons test compared mean *post hoc* differences in physiological outcomes at pre and postlavage baseline and at each time point following treatment between the different groups. The criterion for significance was established *a priori* as *p* < 0.05 for all comparisons. The two additional measurements at 10 and 15 minutes in the D3-MSD2 aerosol animals were descriptive and not included in the statistical analysis, based on a lack of data in the sham and Curosurf liquid historical controls.^11^

## Results

### Spray drying and powder formulation particle sizing

Over the course of the study, we produced multiple batches of the SLS-EEG MSD2 formulation using the Buchi S-300 Mini Spray Dryer system. The spray rate of five batches of MSD2 powder formulation was ∼2.1 mL/min. The DPPC content (mean ± SD) of these five batches assessed using the previously validated HPLC-UV method was 55.5 ± 2.9%w/w, which was 101.1% of the nominal content (55.0%). The B-YL content (mean ± SD) assessed over the same five batches using a validated HPLC-UV method was 2.5 ± 0.1%w/w, which was 100.7% of the nominal content (2.5%). The coefficient of variation for DPPC and B-YL content was ∼5%, indicating good reproducibility in key components across batches.

Table 1 provides the volumetric particle size of the MSD2 formulation determined using the Sympatec laser diffraction technique at dispersion pressures of 0.5 and 4.0 bar, tested across five different batches. The primary particle size (Dv_50_) of the MSD2 formulation batches measured at 4.0 bar dispersion pressure was 1.2 µm with coefficient of variation 2.1%, reflecting the consistent size of the formulation for the five batches considered and reproducibility of the powder preparation process. The D_v50_ (mean ± SD) of the batches measured at 0.5 bar dispersion pressure was 1.8 ± 0.0 µm, suggesting good dispersibility of the powders. Overall, the spray drying process produces micrometer-sized particles with good dispersibility, which are suitable for the EEG approach and deep lung delivery.

**Table 1.** Dispersion Properties of the SLS-EEG MSD2 Formulation at 4.0 and 0.5 Bar Dispersion Pressures from Sympatec Testing (Data Are Mean ± Standard Deviation of Five Different Batches)

Dispersion pressure	Particle size		
	D_v10_ (µm)	D_v50_ (µm)	D_v90_ (µm)
4.0 bar	0.6 ± 0.0 (3.7%)	1.2 ± 0.0 (2.1%)	2.2 ± 0.1 (3.1%)
0.5 bar	0.7 ± 0.0 (1.4%)	1.8 ± 0.0 (2.5%)	4.3 ± 0.1 (3.0%)

Values in parenthesis represent the coefficient of variation.

SLS-EEG, synthetic lung surfactant–excipient enhanced growth formulation.

### Powder formulation imaging

**FIG. 5.**
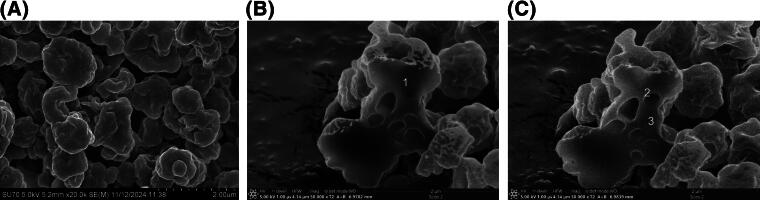
Outer surface and inner structures from FIB/SEM imaging of the SLS-EEG MSD2 particles indicating **(A)** relatively spherical particles with wrinkled outer surfaces and **(B, C)** intermediate size inner hollow voids. FIB sectioning revealed intermediate sized void structures (∼100–300 nm) in most particles ≥1 µm upon sufficient sectioning. Location 1 in Panel B is the start of an addition pore that is revealed at location 2 in Panel C with the next FIB cut of ∼200 nm. Location 3 then indicates the start of a deeper void. FIB, focused ion beam; SEM, scanning electron microscope.

SEM images of particles composing the MSD2 formulation are displayed in Figure 5, at magnifications of 20k and 50k. Imaging of the external particle structure indicated wrinkled surfaces with relatively spherical particle shapes and geometrical diameters consistent with Sympatec volumetric diameter results at 4.0 bar testing (Fig. 5A). FIB sectioning of particles indicated relatively large voids were present in most particles with particle diameters approximately ≥1 µm. As illustrated in Figure 5B, internal voids had diameters of ∼100–300 nm with between-void spacings of ∼100 nm or greater. Smaller openings, as shown at location 1 in Figure 5B, progressed to larger voids as with location 2 in Figure 5C during successive FIB sectioning.

### XRPD and moisture sorption testing

**FIG. 6.**
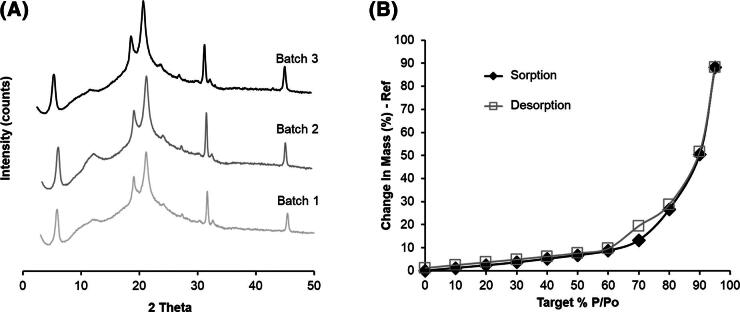
Physicochemical characterization of the SLS-EEG MSD2 formulation from **(A)** X-ray powder diffractograms and **(B)** dynamic vapor sorption (DVS). In Panel b, the particles experience little water mass uptake until >70% relatively humidity (denoted as % P/Po), which is ideal for dry powder EEG aerosol storage and lung targeting.

Figure 6 shows the solid-state properties of the MSD2 powder formulations characterized using XRPD and DVS. The X-ray powder diffractograms of three different batches of the MSD2 formulations (Fig. 6A) showed evidence of a partially crystalline structure with no differences in the peak diffractograms indicating reproducible production of different batches of powders. Characteristic peaks were observed for l-leucine and MN as being in the crystalline state.

Figure 6B shows that following an equilibration period at 0% RH, water uptake by the powder at 10%–60% RH (black line) was <9%, indicating relatively low uptake for an unprotected (i.e., unpackaged) spray-dried powder. Above 70% RH, the water uptake increased significantly, indicating the hygroscopic nature of the formulation at elevated RH, which is well suited for the EEG dry powder aerosol application. Low water uptake during storage under ambient conditions and elevated water uptake at higher humidity conditions are important for powder handling and when hygroscopic growth in the humid lung airways is required for EEG particle size increase.

### Aerosol size characterization using cascade impaction

Table 2 provides the aerosol particle sizing of the MSD2 formulation delivered with the D3 device (one actuation per level) and a 30 mg device-loaded formulation mass based on cascade impaction with the NGI. The device retention (as measured by DPPC assay) was 22.6% with a preseparator deposition fraction of 5%. The low preseparator fraction led to a FPF <5 µm (FPF_<5 µm_) of 87.9%, which was more than four times higher than the previously reported FPF_<5 µm_ (19.3%) for the D2-NSD1 combination.^48^ The mean FPF_<2.5 µm_ was 61.6% after four actuations, suggesting the potential to deliver a substantial amount of powder to the alveolar region. The MMAD of the MSD2 formulation delivered from the D3 device was 2.0 µm, which is lower than the previously reported D2-NSD1 combination of 2.9 µm,^48^ suggesting more efficient aerosolization and better aerosol quality using the new D3-MSD2 combination.

**Table 2.** Aerosol Size Distribution of the SLS-EEG MSD2 Formulation Determined by Using Next-Generation Impactor After Delivering 30 mg Loaded Dose from the D3 Device with One Actuation per Level (Data Are Means ± Standard Deviations, *n* = 3)

Sections and terms	Values
Device retention (%)	22.6 ± 2.1
Preseparator fraction (%)	5.0 ± 2.0
MMAD (µm)	2.0 ± 0.0
FPF_<1 µm_ (%)	10.0 ± 0.6
FPF_<2.5 µm_ (%)	61.6 ± 0.2
FPF_<5 µm_ (%)	87.9 ± 3.3
Emitted dose (%)	77.4 ± 2.1
Total recovery (%)	98.7 ± 3.8

FPF, fine particle fraction; MMAD, mass median aerodynamic diameter.

### Characterization by actuation from SprayVIEW, Spraytec, and emitted mass

**FIG. 7.**
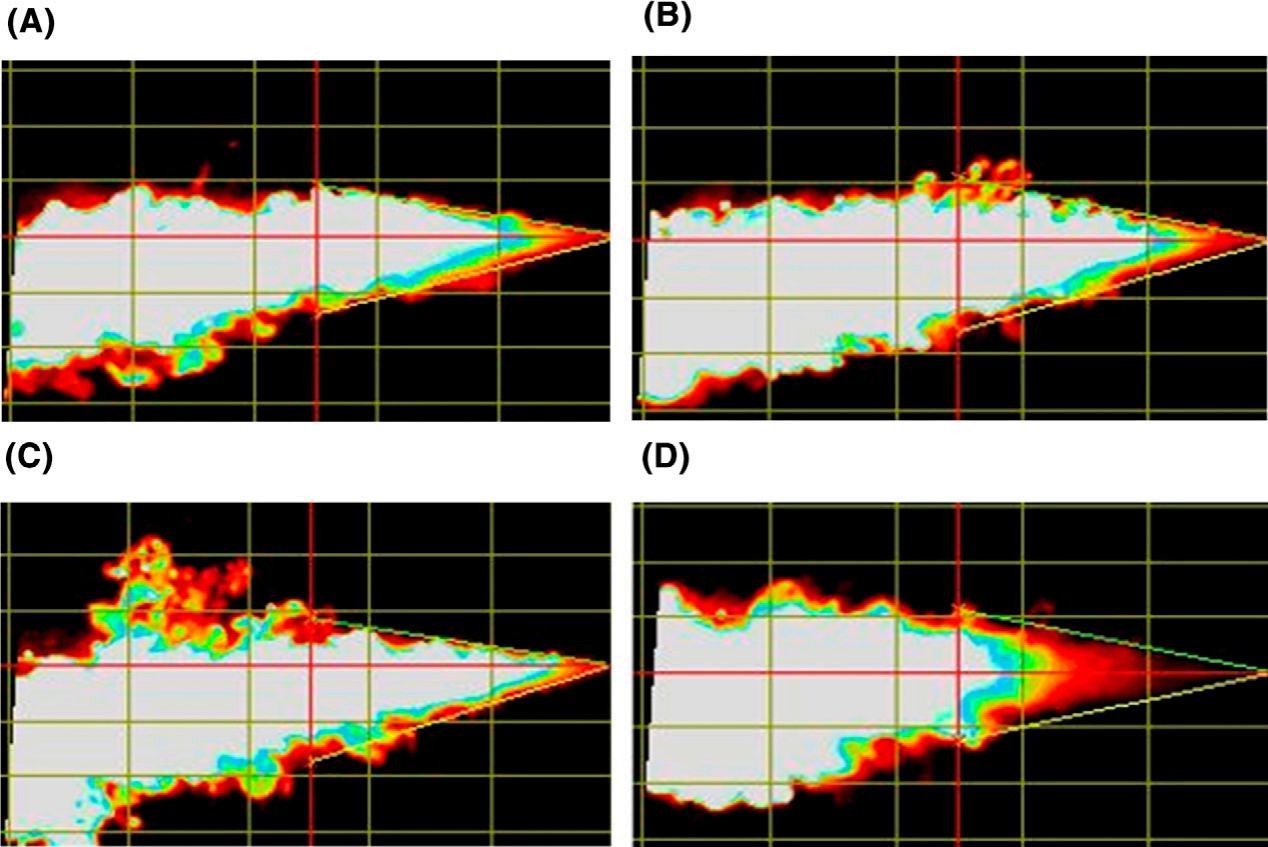
Maximum intensity plume images for successive actuations of the spray (taken ∼50 msec after actuation when the leading plume edge has reached ∼100 mm from the tip of the orifice) with the SprayVIEW® system indicating qualitatively consistent emitted dose over each of the four actuations (A1–A4) during a single experimental run (R1) with 30 mg mass loading: **(A)** A1, **(B)** A2, **(C)** A3, and **(D)** A4. The red vertical line indicates 50 mm from the tip of the orifice (origin; where cone angle lines originate) where the cone angles and widths are taken; the red horizontal line indicates the midline as an angle bisector. Scale is 20 × 10 mm (L × H).

Figure 7 shows a snapshot of the plume geometry of powder aerosol emitted from the D3 device over four actuations. Mean cone angles of plumes during their maximum signal intensity (∼50 ms from start of actuation and ∼100 mm from tip of the device orifice) for actuations A1–A4 were 26.6°, 29.2°, 30.7°, and 27.1°, respectively, with a 1.9° SD across actuations; corresponding mean cone widths for actuations A1–A4 were 23.7, 26.2, 27.5, and 24.1 mm, respectively, with a 1.8 mm SD across actuations. The plume front velocity for the leading edge of the emitted aerosol cloud up to 100 mm from the orifice for each actuation A1–A4 was 3.0, 1.5, 1.9, and 2.0 mm/ms, respectively, with a 0.6 mm/ms SD across actuations. Plume durations (PDs) measured with the Spraytec analysis for actuations A1–A4 were 319, 315, 313, and 270 ms, respectively, with an SD of 22.8 ms. For comparison, the PD observed in SprayVIEW was 10%–20% longer than with Spraytec. Spraytec PD measurements were expected to be more accurate, as the plume enters and exits the measurement zone with minimum recirculation when the detector tower flanges are removed.

**FIG. 8.**
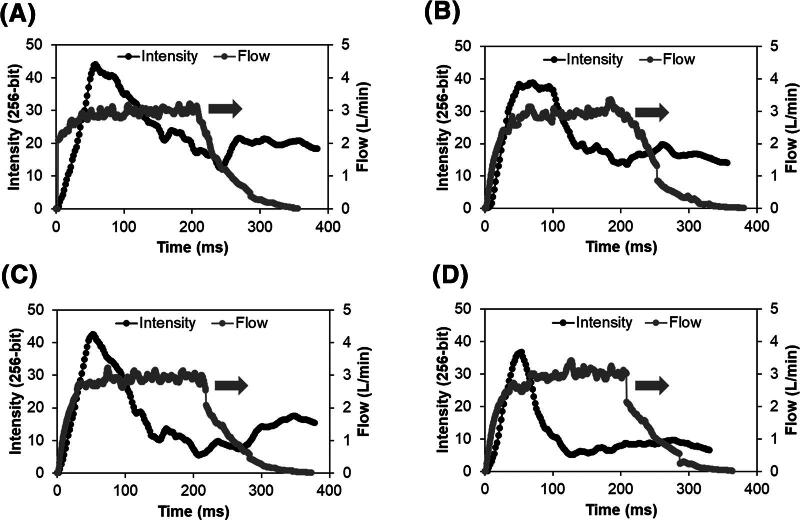
Representative graphical output for plume intensity (black line, left axis; SprayVIEW), and gas flow (gray line, right axis; flow meter) for each device actuation of the D3-MSD2 device and formulation combination when delivering a 30 mg loaded mass during a single experimental run (R1) and using one actuation per turn: **(A)** A1, **(B)** A2, **(C)** A3, **(D)** A4.

A comparison of simultaneous aerosol emission versus device actuation flow rate over time based on SprayVIEW testing for actuations A1–A4 is provided in Figure 8. SprayVIEW intensity measurements were used to approximate aerosol emission over time and selected as a conservatively longer option compared with the Spraytec measurement of obscuration (100-%Transmission). Flow profiles indicated an airflow actuation duration of ∼0.21 seconds, as expected, as well as an asymptotically decreasing flow through ∼0.3 seconds, likely due to compressible flow effects within the D3 device. Interestingly, for all actuations, a majority of the aerosol plume intensity, approximating powder emission, occurred during the first half of the actuation (∼100 ms). This relationship provides evidence that the D3 device is capable of controlling the amount of emitted mass such that a majority of the aerosol enters the first half of the inhalation volume.

**FIG. 9.**
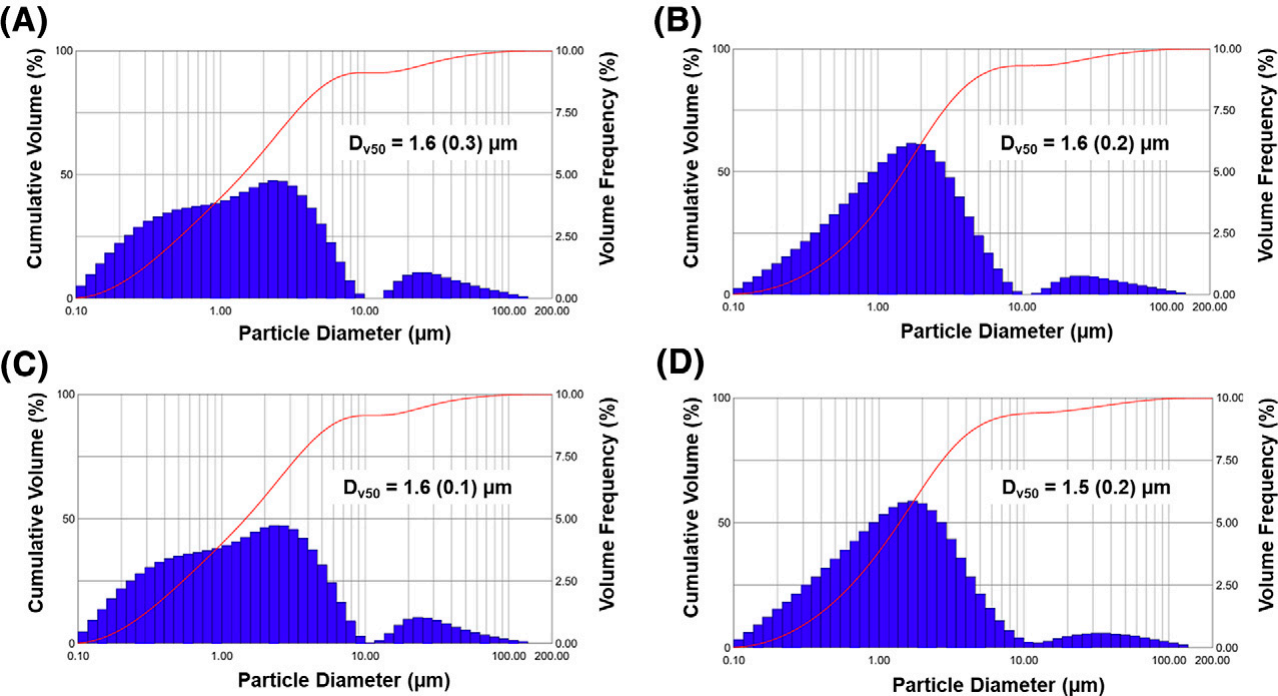
Representative Spraytec® laser diffraction particle size distributions for each device actuation of the D3-MSD2 device and formulation combination when delivering a 30 mg loaded mass during a single experimental run (R1) and using one actuation per turn: **(A)** A1, **(B)** A2, **(C)** A3, and **(D)** A4. Reported Dv_50_ values represent the mean (standard deviation; SD) of the data for each actuation number evaluated over three runs (*n* = 3).

Spraytec evaluation of aerosol size distributions for individual actuations A1–A4 over a single run is illustrated in Figure 9. In each case, a majority of the aerosol volume occurs in particles with geometric diameters <2 µm, and very little volume (∼10%) occurred in particles with geometric diameters >10 µm. The volumetric PSD also appears consistent across the four actuations. Mean ± SD Dv_50_ values evaluated for each actuation A1–A4 resulted in a narrow range of 1.5 ± 0.2 µm to 1.6 ± 0.3 µm. Furthermore, Spraytec laser diffraction FPF values <2.5 µm based on volumetric diameter for actuations 1–4 were 64%, 68%, 65%, and 71%, respectively. In summary, both the mean geometric aerosol size and the particle size distribution were very consistent across the four actuations of the D3 device loaded with 30 mg of the MSD2 formulation and similar to cascade impaction aerodynamic sizing (Table 2).

**FIG. 10.**
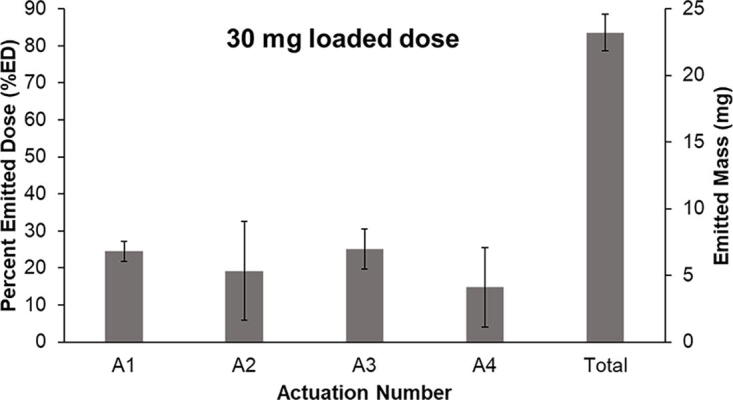
Percent emitted dose (%ED) and emitted mass (milligrams) for each device actuation (A1–A4) of the D3-MSD2 device and formulation combination when delivering a 30 mg loaded mass and using one actuation per turn. Bars represent mean values evaluated over three runs (*n* = 3) and error bars are standard deviations. Total represents the net emission of all four actuations.

Percent emitted dose (%ED) and emitted mass are provided in Figure 10 for actuations A1–A4 and as the sum of all actuations (“Total”). Relatively high and consistent emitted masses within the targeted range of 5–10 mg/actuation were observed over actuations A1–A3. Emitted mass with A4 was just below the target range (4.1 mg); however, this was viewed as acceptable as the final A4 actuation may be considered a final chamber emptying step. Variability and low emission in actuations A1–A3 were typically compensated for with higher emissions in the subsequent actuations, resulting in low overall variability on total emitted mass. The *Total* mean ± SD %ED was 76.7 ± 4.6%, which agreed well with the NGI findings (77.4% ± 2.1; Table 2).

### In vitro testing of lung delivery efficiency with the LC model

Table 3 provides the *in vitro* regional deposition fractions and estimated rabbit lung delivery efficiencies of the D3-MSD2 combination assessed using the *in vitro* LC model when administering a device-loaded formulation mass of 30 mg. The ED from the D3 device was 70.0%, with lower lung (filter + LC) and total lung (TB + filter + LC) delivery efficiencies of 40.1% and 56.8%, respectively, indicating a high fraction of the loaded dose was delivered to the simulated rabbit lungs. Although the lung delivery efficiency from D3 device was slightly lower than the previously reported D2-NSD2 combination,^48^ higher FPF_<5 µm_ in the NGI study suggests significant improvements in aerosol quality, which will be critical for delivering the aerosol directly to the alveolar region with the D3-MSD2 combination in the *in vivo* experiments.

**Table 3.** Regional Deposition Fractions (%) and Lung Delivery Efficiencies of the SLS-EEG MSD2 Formulation (30 mg Loaded Mass) Delivered from the D3 Device to the *In Vitro* LC Model (Data are Means ± Standard Deviations, *n* = 3)

Sections and terms	PL mass (%)
Air-jet device	30.0 ± 2.7
Endotracheal tube (ETT)	13.4 ± 2.5
Tracheobronchial region (TB)	16.7 ± 0.7
Lung chamber (LC)	30.3 ± 3.2
Filter	9.8 ± 0.4
Lower lung dose (LC + Filter)	40.1 ± 3.3
Total lung dose (TB + LC + Filter)	56.8 ± 2.8
Emitted dose	70.0 ± 2.7
Total recovery	100.2 ± 2.3

### In vivo animal descriptions and dosing

Following lung lavage, the mean PaO_2_ was reduced from ∼460 to 65 mmHg and weight normalized compliance from ∼1.1 to 0.4 mL/(cmH_2_O·kg) at baseline (*p* < 0.001) with increased PaCO_2_ and disease severity (OI >15 and VEI ∼0.03) values, all indicative of severely depleted surfactant pools, elevated alveolar surface tension, and severe RDS. Baseline data following induction of lung lavage for surfactant deficiency were not different between groups for body weight, gas exchange, disease severity, and lung compliance.

All the animals in the liquid Curosurf group (historical controls^11^) experienced some degree of acute airway obstruction from the liquid, resulting in hypoxemia (SpO_2_ < 85%), hemodynamic instability (e.g., bradycardia or hypotension), or increased PIP (>25 cmH_2_O) by the ventilator during dosing. The D3-MSD2 aerosol and control groups experienced acute hypoxemia (SpO_2_ < 85%) upon ventilator disconnection that quickly resolved when positive-pressure actuations were applied for therapy with the iDP-ADS. Following the treatment, the heart rate (*p* = 0.94) and blood pressure (*p* = 0.93) did not vary between the groups.

As described, based on an experimental mean rabbit weight of 1.59 kg, the device-loaded dose of 60 mg of the SLS-EEG MSD2 powder formulation contained 24 mg-PL/kg compared with the approximate 200 mg-PL/kg with Curosurf.^75^ The total treatment duration, which included stabilization, was ∼10 minutes to administer Curosurf. The aerosol delivery required eight actuations for 60 mg delivery of the D3-MSD2 formulation with the D3 device and could be completed in <1 minute. The total therapy delivery time, including ventilator reconnection to enable device reloading and stabilization (i.e., two 30 mg device-loaded dose delivery cycles), was completed in <5 minutes.

### In vivo gas exchange

**FIG. 11.**
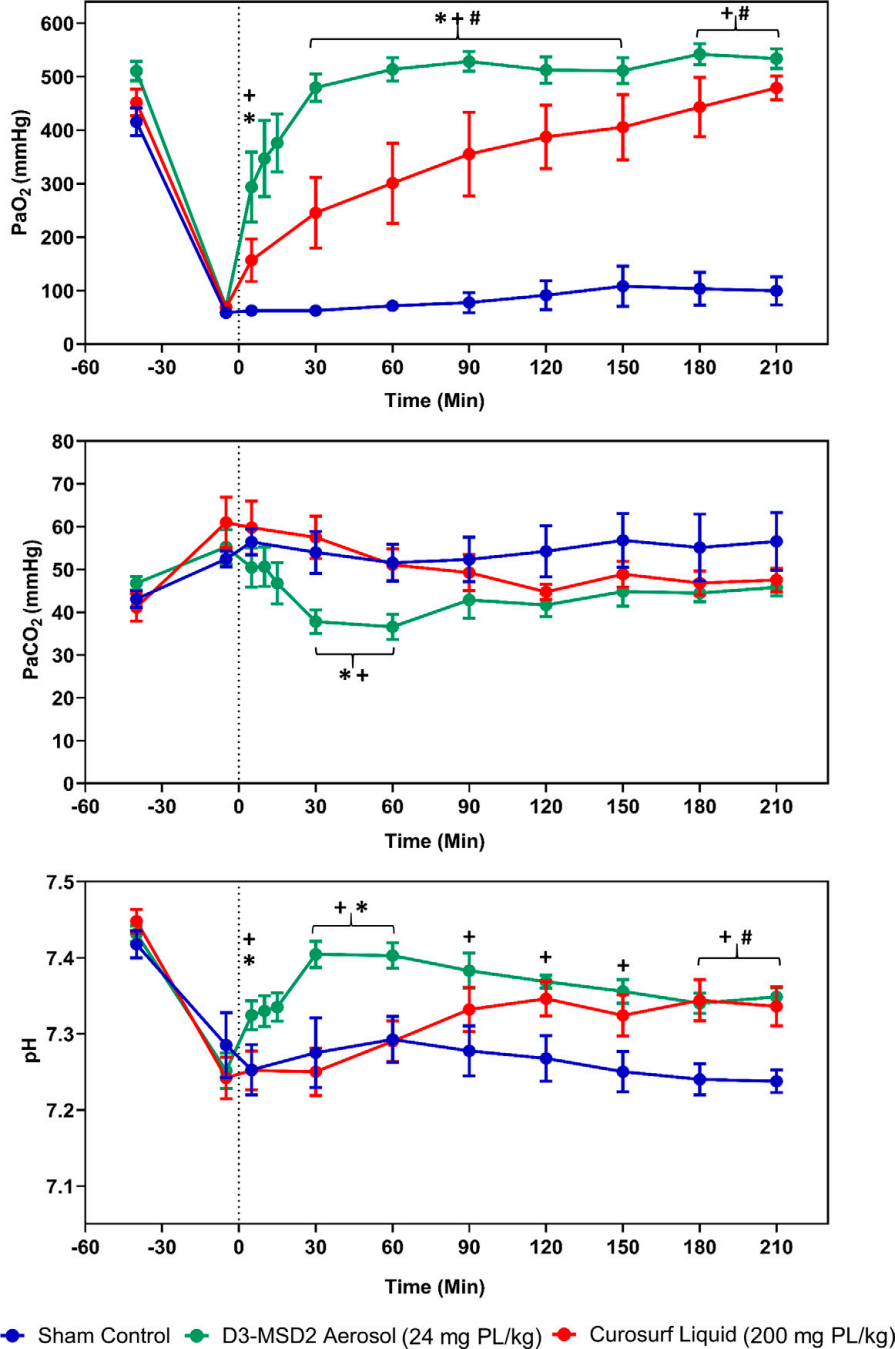
Gas exchange data are shown as mean ± standard error of mean (SEM) for control and surfactant treatment groups during the 210-minute study period. The vertical dashed line represents the time point (0 minutes) following the completion of liquid Curosurf (red circles) administration and the initial 30 mg dose of D3-MSD2 (SLS-EEG) aerosol (green circles) or sham control (blue circles). The mean PaO_2_ was higher in the aerosol treatment group than Curosurf and sham groups. Following the second (final) aerosol (30 mg) and sham deliveries at 15 minutes, the PaO_2_ values between 30 and 150 minutes were greater with aerosol than the Curosurf and control groups. The Curosurf group had higher PaO_2_ than the controls. Among the surfactant groups, arterial oxygenation was not different at 180 and 210 minutes, and PaO_2_ recovered to 94% of the prelavage values with liquid Curosurf and 99% with aerosol at 3.5 hours. The liquid Curosurf PaCO_2_ values were not different from controls during the experiment. The aerosol group had statistically lower PaCO_2_ than the liquid Curosurf and control groups at 30- and 60-minute time points but did not differ between groups following the remainder of the experiment. The aerosol treatment group had greater pH than controls at all time points throughout the experiment. Compared with liquid Curosurf, the aerosol group had greater pH at the 5-, 30-, and 60-minute time points but was not different at the other time points. Two-way ANOVA and post hoc Tukey’s test compared mean differences at each time point between the groups. ^*^p < 0.05 for D3-MSD2 aerosol vs. liquid Curosurf. ^+^*p* < 0.05 for control vs. D3-MSD2 aerosol. ^#^*p* < 0.05 for control vs. liquid Curosurf.

Gas exchange data for the different *in vivo* groups are shown in Figure 11. Following surfactant depletion, all groups had mean PaO_2_ values below 70 mmHg, on FiO_2_ of 1.0 at baseline, indicative of severe RDS. As expected, PaO_2_ values increased from baseline in the surfactant groups immediately following therapy and remained <100 mmHg in the sham control group throughout the experiment. The mean PaO_2_ at the 5-minute time point was not different between the Curosurf and control groups. In contrast, at the 5-minute time point (which included only the first 30 mg device-loaded dose), the D3-MSD2 aerosol group was significantly greater than both the sham control and Curosurf liquid groups, with a PaO_2_ of ∼300 mmHg. Following the second (final) device-loaded dose of 30 mg and delivery at 15 minutes, the PaO_2_ values between 30 and 150 minutes were significantly greater with aerosol than the Curosurf and sham control groups. As expected, the Curosurf group had higher PaO_2_ than the sham control throughout the experiment. Among the surfactant groups, arterial oxygenation was not different at 180 and 210 minutes, and PaO_2_ values recovered to 94% of the prelavage with Curosurf liquid. For the D3-MSD2 combination, PaO_2_ values exceeded the basal levels at 180 minutes and ended with a final PaO_2_ level that was 99% of the prewashout value at 210 minutes.

As expected, the mean PaCO_2_ increased, and pH decreased among groups due to severe RDS induced by lung lavage (Fig. 11). The PaCO_2_ values in controls remained increased throughout the experiment, and although PaCO_2_ trended downward in both surfactant groups, the liquid Curosurf values were not different from sham controls during the experiment. The PaCO_2_ values with the D3-MSD2 aerosol were statistically lower than liquid Curosurf and controls at 30- and 60-minute time points but did not differ from the Curosurf or sham control groups for the remainder of the experiment. The mean pH values remained between 7.25 and 7.30 in the controls and remained low but were no different from liquid Curosurf, except for the 180 and 210-minute time points, where Curosurf pH was higher than control. The aerosol treatment group had greater pH than controls at all time points throughout the experiment, beginning with the 5-minute blood gas. Compared with liquid Curosurf, the aerosol group had greater pH at the 5-, 30-, and 60-minute time points but was not statistically different at the remaining time points (see Fig. 11), despite having a higher trend at all time points.

### In vivo lung compliance and disease severity

**FIG. 12.**
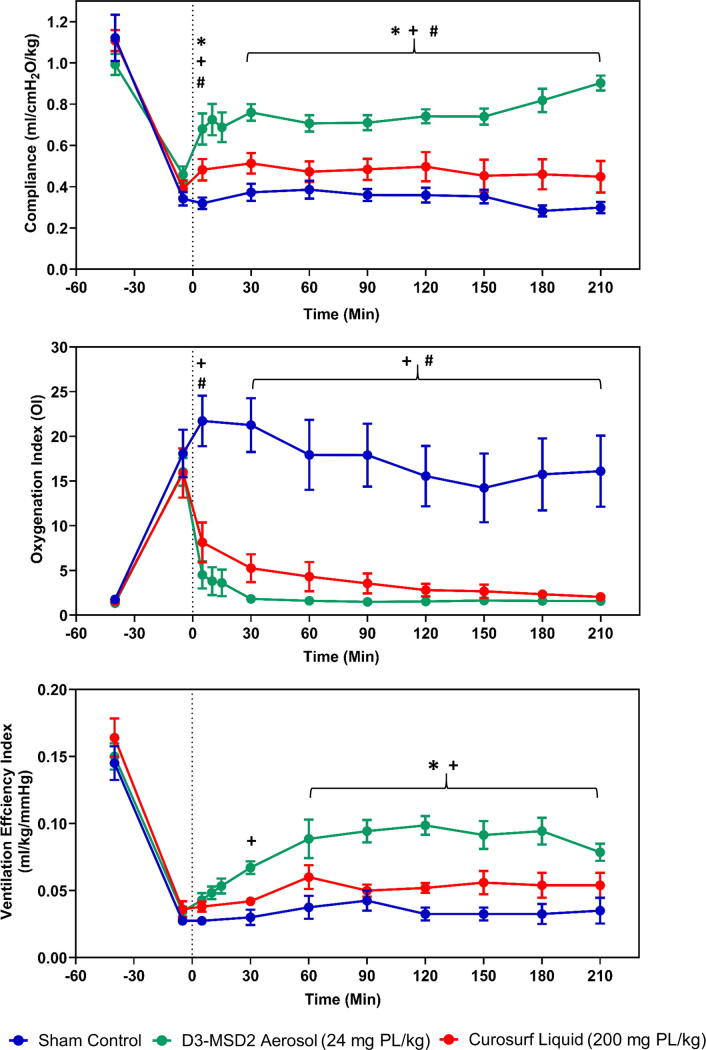
Lung compliance, oxygenation index (OI), and ventilation efficiency index (VEI) data are shown as mean ± SEM for control and surfactant treatment groups during the 210-minute study period. The vertical dashed line represents the time point (0 minutes) following the completion of liquid Curosurf (red circles) administration and the initial 30 mg dose of D3-MSD2 (SLS-EEG) aerosol (green circles) or sham control (blue circles). The lung compliance improved rapidly in the aerosol group 5 minutes following the initial 30 mg surfactant dose and continued to improve following the 30 mg aerosol delivery at 15 minutes. The aerosol group had greater compliance than the liquid Curosurf and control groups from the 5-minute time point until the end of the experiments. At the 3.5-hour time, the compliance in the liquid Curosurf and sham control groups was not improved from baseline, but surfactant D3-MSD2 compliance approximately doubled compared with washout baseline and recovered to 91% of the prewashout value. The VEI was higher in the D3-MSD2 aerosol surfactant group than the controls at 30 minutes, and both surfactant treatment groups had higher VEI than the controls from 60 minutes until the 3.5-hour observation period. Two-way ANOVA and post hoc Tukey’s test compared mean differences at each time point between the groups. ^*^p < 0.05 for D3-MSD2 aerosol vs. liquid Curosurf. ^+^*p* < 0.05 for control vs D3-MSD2 aerosol. ^#^*p* < 0.05 for control vs liquid Curosurf.

Lung compliance and indices of disease severity data for the different groups are shown in Figure 12. The lung compliance improved rapidly in the aerosol group at 5 minutes following the initial 30 mg device-loaded dose and continued to improve following the second 30 mg aerosol delivery at 15 minutes. The D3-MSD2 aerosol group had greater compliance than the liquid Curosurf and control groups from the 5-minute time point until the end of the experiments. Following a small initial increase between 5 and 30 minutes, the compliance values stabilized and then slightly decreased with liquid Curosurf and were not statistically different from sham controls at any of the time points over the 3.5-hour observation period. At the 3.5-hour final time, the compliance in the liquid Curosurf and sham control groups was not improved (0.4 vs. 0.3 mL/[cmH_2_O·kg], respectively) from baseline. In contrast, the D3-MSD2 aerosol group compliance nearly doubled from baseline (0.46–0.91 mL/[cmH_2_O·kg]) and recovered to 91% of the prewashout value. Furthermore, the D3-MSD2 aerosol group demonstrated an increasing trend in compliance through the final hour of the experiment that continued to the 3.5-hour final time point.

Surfactant washout produced OI >15 for all cases, consistent with severe respiratory disease. As expected, improvement was not observed for the sham control case over the 3.5-hour observation period, and OI values were higher than the surfactant-treated groups at all time points. In contrast, the surfactant aerosol group showed a rapid reduction in OI (<2) with 80%–91% recovery from baseline (prelavage) that was maintained from the 30-minute time point until the end of the 3.5-hour period. The liquid Curosurf group showed a favorable reduction in OI following treatment with a more gradual recovery, ranging from 28% to 65% between 30 and 180 minutes and 75% at 3.5 hours.

Considering VEI, liquid Curosurf demonstrated an improved trend compared with the sham control throughout the observation period that did not rise to the level of statistical significance. In contrast, the D3-MSD2 aerosol group demonstrated a statically significant improvement versus the sham control at 30 minutes, which continued through the 3.5-hour experiment. Furthermore, the D3-MSD2 aerosol group demonstrated statistically significant improvement versus liquid Curosurf at 60 minutes, which also continued through the remainder of the 3.5-hour observation period.

## Discussion

AST offers a number of theoretical advantages that may ultimately include the avoidance of intubation for lung surfactant administration, elimination of side effects associated with intubation and surfactant liquid bolus instillation, improved efficacy at reduced doses (with associated reduced costs), and the ability to administer the therapy repeatedly as needed, possibly as a preventative measure, due to a low-risk and high-reward therapeutic profile.^1,3,4^ Despite these potential advantages, as described in the Introduction, typical limitations of AST in previous animal models and human subject trials in comparison with the current clinical practice of liquid bolus instillation through an ETT or intubation catheter include (1) higher administered dose for an adequate therapeutic response, (2) longer delivery times, (3) equivalent or slower response times, and (4) muted biological response. Our group is developing a dry powder aerosol SLS product with the goal of efficient delivery of a highly active synthetic formulation directly to the alveolar region. In a recent two-part study, we demonstrated that a combination of a D2 AJ DPI and SLS-EEG NSD1 formulation addressed items (1) and (2) by achieving equivalent to superior biological response compared with liquid bolus instillation at an order of magnitude-reduced PL dose. In this study, we have provided *in vivo* animal model evidence, for the first time, that an aerosol therapy can surpass the performance of liquid bolus instillation in all four areas, and in most cases with order of magnitude improvements, as further described below.

Considering the administered dose of the aerosol lung surfactant product, the total device-loaded dose of active PL components (combination of DPPC and POPG) was 24 mg/kg. Based on *in vitro* testing in the NGI, device %ED was 77.4%, resulting in a device ED of 18 mg-PL/kg, which was administered directly to the ETT. In comparison, Curosurf was administered at a clinically recommended first dose of 200 mg/kg and consisted almost entirely of PL components. Furthermore, based on high PL and surfactant protein content, Curosurf is often the preferred clinical option for surfactant replacement therapy in RDS.^5,25^ Despite the order of magnitude lower administered PL dose associated with the aerosol, the AST met or exceeded response to Curosurf for every metric tested. Furthermore, the AST exceeded Curosurf performance at every time point, often significantly, with the exception of pH for the final two time points where performance largely overlapped.

We suspect superior performance of the aerosol at order-of-magnitude reduced doses was possible due to improved delivery of a small particle formulation directly to the alveolar region. Liquid bolus instillation is expected to be associated with a significant “coating cost” in the TB airways, such that alveolar delivery is a low percentage of the instilled dose.^38,76^ Furthermore, liquid bolus instillation is often associated with blockage of the ETT, significant surfactant reflux, clogging of the trachea, oxygen desaturation, and additional alveolar injury.^4,7–11,27,77^ In contrast, the aerosol therapy showed no evidence of airway blockage and, we expect, improved delivery of the active components directly to the alveolar region.

Considering the time required for therapeutic aerosol delivery, previous animal and human subject AST studies reported aerosol administration times of 15 minutes to 2.5 hours, with multiple delivery cycles often required.^19,26,78^ For administration of the D3-MSD2 combination in this study, two dose delivery cycles were employed, each requiring an aerosol delivery time of ∼20 seconds. Total aerosol delivery time with the D3-MSD2 combination was therefore <1 minute. In practice, animal care together with disconnection and reconnection with the ventilator required a total therapy delivery time of <5 minutes, which could also be reduced to <1 minute by better integration of the iDP-ADS with the ventilator.

One of the most striking advantages of the AST in this study was the rapid and almost instantaneous response achieved in the animal model of severe RDS. For example, at the 5-minute blood gas reading when only the first 30 mg device-loaded dose had been administered (12 mg-PL/kg), the mean PaO_2_ was already at ∼300 mmHg. This 5-minute recovery value is already far in excess of the ∼50–80 mmHg value achieved by a recent nebulization study after 15 minutes and ∼200 mmHg at 180 minutes following administration of 100 mg of surfactant^18^ and equal to the final 180-minute value achieved with a previous dry powder therapy.^19^ Similarly, almost instantaneous response to the AST with significant differences at the 5-minute time point (vs. sham control and vs. liquid Curosurf) was observed for the *in vivo* metrics of pH and compliance. PaCO_2_ and VEI with AST then reached the level of statistical difference (vs. sham control and vs. liquid Curosurf) at the 30- and 60-minute time points, respectively. The times required for the D3-MSD2 aerosol therapy and instilled Curosurf liquid to restore oxygenation to 300 mmHg were ∼5 minutes and ∼60 minutes, respectively, resulting in a ∼12-fold faster response with the aerosol therapy. Similar times to reach ∼400 mmHg were 30 and 150 minutes, respectively, resulting in a ∼5-fold faster response with the aerosol. Considering a 500-mmHg metric, the D3-MSD2 aerosol therapy required 60 minutes, whereas the Curosurf liquid did not reach this level. Based on these observations, response rate to the D3-MSD2 therapy appears to be increased by approximately an order of magnitude, compared with both previous aerosol therapies considered and with the current clinical standard of liquid Curosurf administration in the same animal model.

While previous aerosol therapies have reported relatively muted biological response compared with liquid bolus instillation, the response to the D3-MSD2 formulation may be considered dramatic. As described above, PaO_2_ levels responded 5- to 12-fold times faster with aerosol, were significantly higher than with liquid Curosurf for a majority of the experiment (i.e., through 150 minutes), and achieved values >500 mmHg, also not achieved with the liquid. One of the largest differences in therapeutic response was observed in the lung compliance where the D3-MSD2 therapy produces statistically higher values than the sham control and liquid Curosurf at the 5-minute time point and at all subsequent time points (for which comparison data were available). Moreover, compliance of the sham control animals averaged ∼0.35 mL/(cmH_2_O·kg) and liquid Curosurf produced an approximate 43% recovery, compared with the prelavage compliance, to an average value of 0.48 mL/(cmH_2_O·kg). The D3-MSD2 aerosol therapy resulted in an average value of ∼0.78 mL/cmH_2_O/kg and a final value of 0.90 mL/(cmH_2_O·kg). The final compliance recovery with the D3-MSD2 therapy was 91% of the original prelavage value of 0.99 mL/(cmH_2_O·kg). Similarly, the VEI with the D3-MSD2 therapy remained significantly higher than both the control and liquid Curosurf beginning at 60 minutes and continuing through the remainder of the experiment. In summary, the D3-MSD2 therapy appeared biologically superior to liquid Curosurf instillation with strong statistical significance in the rate of oxygenation, PaCO_2_ and pH recovery, and in the rate and final magnitude of compliance and VEI.

The significant improvements in therapeutic response associated with the D3-MSD2 aerosol therapy compared with the previous D2-NSD1 combination are most likely associated with improvements in the aerosol quality and delivery protocol, leading to higher mass of active surfactant components delivered deeper within the lungs and possibly all the way to the alveolar region using positive-pressure actuations. Based on NGI testing, the previous D2-NSD1 combination produced FPFs (as a % of ED) with cutoffs of <5, <2.5, and <1 µm of 27%, 21%, and 9%, respectively. With the new D3-MSD2 combination, where the D3 device enabled increased dose loading and better regulated %ED/actuation, corresponding FPFs were 88%, 62%, and 10%, respectively. A target FPF (or particle size range) intended to maximize the delivery of EEG particles to the alveolar region in a preterm neonate is currently unknown and awaits ongoing computational fluid dynamics (CFD) simulations. However, we previously conducted numerical simulations in terms of infants with an ETT interface and estimated that an aerosol size without EEG growth of 2.5 µm provided an optimal delivery to the alveolar region, taking into account upstream deposition and the potential for aerosol exhalation. Implementing a FPF_<2.5 µm_ as the best available particle size cutoff metric, as well as the NGI testing data from Table 2, the previous D2-NSD1 combination administered a total fine particle dose (FPD) <2.5 µm of 4 mg-PL/kg, and the current D3-MSD2 therapy administered a FPD_<2.5 µm_ of 11 mg-PL/kg. In terms of aerosol quality, evaluated by FPF, the new D3 device increased the FPF_<2.5 µm_ by a factor >3-fold. While the D3 device had a slightly reduced total %ED, the D3-MSD2 therapy also improved FPD_<2.5 µm_ by ∼3-fold. Additional aerosol delivery advantages provided by the D3-MSD2 combination included the ability to load 30 mg of powder formulation at once, near-elimination of a significant fraction of particle aggregates (captured in the NGI preseparator and likely depositing in the upper TB region), frontloading of the aerosol into the first ∼1/2 of the inhalation waveform, and unifying the delivery across multiple actuations.

Based on the previous *in vitro* testing of Momin et al.,^48^ theoretical considerations of surfactant pool size, and the previous results of Kamga et al.,^12^ a DDP was constructed for dry powder aerosol lung surfactant therapy, which resulted in a high-efficacy response in the subsequent study of DiBlasi et al.^11^ Components of this DDP included^48^

- ∼15 mg-PL/kg delivered to the lungs, as predicted by *in vitro* LC model testing and
- FPD with <1 and <5 µm cutoffs of ≥2 and ≥5 mg-PL/kg based on NGI testing.

In the current study, the D3-MSD2 combination approximates or exceeds these requirements, as described above. Moreover, to better refine the DDP with the goal of a high-efficacy and rapid response, we recommend the addition of a FPD_<2.5 µm_ metric. As described above, the previous study of Momin et al.^48^ had a FPD_<2.5 µm_ of 4 mg-PL/kg. The current study provided a FPD_<2.5 µm_ of 11 mg-PL/kg with a full 60 mg total device-loaded formulation mass. However, a dramatic response and recovery rate were observed after the first 30 mg device-loaded dose, containing a FPD_<2.5 µm_ of only 5.5 mg-PL/kg. Therefore, as an additional DDP metric in a rabbit washout model of severe RDS, we recommend

- FPD_<2.5 µm_ ≥5–10 mg-PL/kg, preferably administered with positive-pressure inflations with a maximum lung pressure of ∼20–25 cmH_2_O.

While we expect improvement in aerosol quality played a predominate role in the improved performance of the D3-MSD2 aerosol therapy observed in this study, there were multiple other aspects of aerosol delivery that we also expect to be important for achieving a rapid high-efficacy response and should also be considered part of the target DDP. First, EEG of the aerosol likely minimized the exhaled dose fraction, despite the small aerosol size. During *in vivo* testing, very little aerosol was observed in the exhaled breath. Even with EEG growth, it is possible to make the initial aerosol size too small. Improved estimates are currently needed, perhaps through complete-airway CFD modeling, for a target EEG initial aerosol size range, which we expect to be ∼0.9–2.0 µm with growth at current excipient levels to ∼2.0–4.4 µm within the lungs. Frontloading of the aerosol into the first half of inhalation was also previously shown to be important by Longest et al.^45^ and was demonstrated with a dry powder aerosol in the current study. Rapid delivery of the aerosol, to minimize the time that the recipient is exposed to ventilation pressures without surfactant, likely resulted in less lung damage, as previously illustrated in DiBlasi et al.^11^ The small primary particles of the aerosol likely helped to form an initial thin surface coating of surfactant over the large surface area of the alveolar region. The presence of hygroscopic excipients may have also helped to dissipate and spread the surfactant components upon deposition at the micrometer level. Finally, administration with positive-pressure inflations is expected to better open collapsed lung regions, which is a common goal in ventilation therapy, and these lung regions are then held open by the locally deposited and quickly spreading surfactant droplets.

A primary limitation of the current study is aerosol delivery through an ETT, which does not realize the full noninvasive potential of AST. Despite CPAP and NIV being the favored clinical approaches for all infants,^5,79,80^ a significant proportion (often >40%)^81,82^ of these infants will fail NIV and/or CPAP support and require invasive ventilation. The current iDP-ADS with the ETT adaptor can be used directly in these scenarios. However, to effectively deliver the aerosol through the nose and to the lungs consistent with CPAP or NIV therapy, additional development work is needed. We have recently demonstrated that EEG aerosols can be efficiently administered to preterm infants transnasally.^52,53,61,68,83^ Integration of these interfaces and techniques with the iDP-ADS and SLS-EEG formulations is currently underway.

The surfactant washout rabbit model provided multiple benefits including consistent body weight with a preterm infant and a multiple-hit injury approach from which the control animals did not recover over the time course of the experiments. One limitation of this model was that the alveolar structure of juvenile rabbits is largely mature. In contrast, depending on the GA of a preterm infant, the alveolar structure is still forming and may lack full septation and volume-filling. In addition, the amount of surfactant remaining in the lungs after washout was not fully quantified. A small amount of surfactant likely remains, potentially including trace amounts of surfactant proteins B and C.

A further limitation of the *in vivo* model may be the use of FiO_2_ of 1.0 throughout the experiment. As described, this nonclinical level of hyperoxia is intended to standardize oxygen therapy and ensure adequate oxygenation and prevent deterioration despite potential complications that could arise during therapy, cause additional lung injury, and prevent the formation of endogenous surfactant. However, in the current study, the animals recovered so quickly in the aerosol group that it was challenging to reduce the ventilation rate and ventilation support fast enough to avoid the deleterious effects of hyperinflation and increased alveolar dead space due to the rapid improvements in lung compliance. Based on the rapid recovery of the animals, we envision future studies in which we evaluate the potential to extubate and discontinue mechanical ventilation shortly after surfactant administration.

## Conclusions

This study has demonstrated that a high efficiency AST approach can significantly exceed the performance of the current clinical standard of high-volume liquid bolus surfactant instillation in an animal model of severe RDS across the critical areas of (1) dose administered, (2) delivery time, (3) response rate, and (4) response magnitude. The D3-MSD2 aerosol therapy enabled an order of magnitude reduction in administration of the active component (PL) dose compared with liquid bolus instillation of the clinically favored animal-derived Curosurf product while achieving an equivalent or, for some metrics, superior response. At the end of the experimental period, the AST produced significant improvements in comparison with liquid Curosurf for the *in vivo* metrics of compliance and VEI. The response rate to the AST was also dramatic, as exemplified by a 5- to 12-fold faster recovery of oxygenation compared with Curosurf. These recovery rates and compliance values were also superior to a recent SLS-EEG surfactant aerosol study employing our previous D2-NSD1 combination.^11,48^ It is expected that the high-efficacy response and rapid recovery rate are largely due to further improvements in the aerosol quality, better enabling the targeting of small-particle aerosol directly to the alveolar region. Comparisons between the *in vitro* testing and *in vivo* response lead to an additional DDP metric for severe RDS of FPD_<2.5 µm_ ≥5–10 mg-PL/kg, determined with NGI testing. Additional advantages of the D3-MSD2 aerosol therapy administered with the iDP-ADS that may have contributed to a high-efficacy rapid response and should be considered in the recommended DDP include:

- Use of the EEG aerosol delivery strategy,
- Frontloading the aerosol delivery into the first portion of the inhalation waveform,
- Small primary particles (e.g., ∼ 1 µm) containing some (e.g., 15% w/w) hygroscopic material, and
- Administration of the aerosol with positive-pressure breaths.

In future studies, we plan to further explore the impact of formulation content and the role of the surfactant protein mimic, B-YL, include the option for extubation of the animal model, and develop systems for transnasal surfactant aerosol delivery for noninvasive administration with and without CPAP support.

## Data Availability

The datasets generated and evaluated during the current study are not publicly available but are available from the corresponding author upon reasonable request.

## Declarations

All experimental animal procedures were conducted according to the National Institute of Health’s Guide for the Care and Use of Laboratory Animals and approved by the Seattle Children’s Institutional Animal Care and Use Committee (ACU00693). The authors W.L. and M.H. are members of the *Journal of Aerosol Medicine and Pulmonary Drug Delivery* Editorial Board.

## Abbreviations Used

AJ: air-jet
AST: aerosol surfactant therapy
DDP: dose delivery profile
DPI: dry powder inhaler
DPPC: dipalmitoyl phosphatidylcholine
D_V50_: volumetric median diameter
DVS: dynamic vapor sorption
%ED: percent emitted dose
EEG: excipient enhanced growth
EM: electromechanical
ETT: endotracheal tube
FIB: focused ion beam
FPD: fine particle dose
FPF: fine particle fraction
GAV: gas actuation volume
HPLC: high-performance liquid chromatography
iDP-ADS: infant dry powder aerosol delivery system
LC: lung chamber
MMAD: mass median aerodynamic diameter
MN: d-mannitol
MSD2: mini spray dryer formulation #2
NGI: next-generation impactor
NIV: noninvasive ventilation
NSD1: nano spray dryer formulation #1
OI: oxygenation index
PEEP: positive end expiratory pressure
PIP: peak inspiratory pressure
PL: phospholipid
PMC: pressure monitoring and control
POPG: 1-palmitoyl-2-oleoyl-sn-glycero-3-phospho-(1′-rac-glycerol) (sodium salt)
PSD: particle size distribution
RDS: respiratory distress syndrome
RH: relative humidity
SEM: scanning electron microscope
SD: spray dried or standard deviation
SLS: synthetic lung surfactant
TB: tracheobronchial
UV: ultraviolent
VEI: ventilation efficiency index
XRPD: X-ray powder diffraction
